# Leg amputation modifies coordinated activation of the middle leg muscles in the cricket *Gryllus bimaculatus*

**DOI:** 10.1038/s41598-020-79319-6

**Published:** 2021-01-14

**Authors:** Dai Owaki, Hitoshi Aonuma, Yasuhiro Sugimoto, Akio Ishiguro

**Affiliations:** 1 Department of Robotics, Graduate School of Engineering, Tohoku University, Sendai, 980-8579 Japan; 2 Research Institute for Electronic Science, Hokkaido University, Sapporo, 060-0812 Japan; 3 Department of Mechanical Engineering, Osaka University, Suita, 565-0871 Japan; 4 Research Institute of Electrical Communication, Tohoku University, Sendai, 980-8577 Japan

**Keywords:** Animal behaviour, Electromyography - EMG

## Abstract

Insects alter their walking pattern in order to respond to demands of an ever-changing environment, such as varying ground surface textures. They also exhibit resilient and flexible ability to retain the capacity to walk even after substantial changes in their body properties, e.g. leg amputation. While the motor control paradigm governing the inter-leg coordination in such adaptive walking has been extensively described in past studies, the mechanism remains unknown. Here, we examined this question by using the cricket (*Gryllus bimaculatus*), which shows a tetrapod/tripod gait on a flat surfaces, like many other insects. We performed leg amputation experiments to investigate modifications of leg movements and coordination of muscle activities. We simultaneously recorded (1) the leg movements, locomotion velocity, and body rotation and (2) the leg movements and leg muscles activities before and after leg amputation. Crickets displayed adaptive coordination of leg movement patterns in response to amputations. The activation timings of levator muscles in both middle legs tended to synchronize in phase when both legs were amputated at the coxatrochanteral joint. This supports the hypothesis that an intrinsic contralateral connection within the mesothoracic ganglion exists, and that mechanosensory feedback from the legs override this connection, resulting in the anti-phase movement of a normal gait.

## Introduction

Insects have evolved many sophisticated behaviours which have enabled them to spread widely and become the most abundant animal group on Earth. One of the most crucial and fundamental functions that promotes insect fitness is locomotion. Through locomotion, insects are able to explore and find resources such as food, mating partners, optimal habitats for laying eggs, and so on. Although insects may sustain various damages, such as to their appendages, from dangerous encounters with their environments, motility continues to be central to the survival of most species. The loss of an appendage changes body balance of insects, thus altering ordinary walking movements. However, some insects have been shown to continue to exhibit resilient and flexible locomotion despite a drastic change in their body structure, e.g. leg amputation^1–3^. In this study, we seek to understand the mechanisms underlying the resilience and flexibility of insect locomotion by considering the cricket (*Gryllus bimaculatus*), which is frequently used to investigate topics as varied as locomotion, auditory taxis^4–6^, aggressive behavior^7–9^, learning and memory^10–12^, etc. Crickets, along with many other insects^13–15^, show a well-coordinated tetrapod/tripod walking pattern on flat ground. Previous studies have suggested that the inter-leg coordination of locomotion is controlled in part by distributed neural networks located in the thoracic ganglia known as leg central pattern generators (leg CPGs)^16–21^. The coordinated patterns are modulated by a higher motor centre, the subesophageal ganglion (SEG)^22,23^. Although it is also known that sensory feedback affects leg coordination^24–26^, it remains unclear how insects enact adaptive walking patterns after losing one or more legs. This aspect has been the focus of study of several biologists as well as robotics engineers, who have attempted to develop multi-legged robots that can display adaptive locomotion even if they lose one or more legs. With the advancement in technology, a high-performance CPU has aided in controlling the movement of robots; however, the complex and adaptive behaviours of insects can be exhibited only negligibly by the robots. The insect nervous system is comprised of approximately $10^5$ to $10^6$)^27,28^ neurons; hence, it is necessary to take into account the role of the intrinsic neural circuits that influence the adaptations of insects under unfavourable circumstance, as well as the sensory feedback mechanisms, which reflect their body characteristics and physical interactions with the environment. Therefore, it is essential to clarify the mechanisms underlying adaptive locomotion, which will be beneficial to the fields of both biology and robotics, and aid in designing robust and resilient multi-legged robots that can adapt to physical damage similar to insects.

One useful approach to elucidating the adaptive inter-leg coordination mechanism that insects recourse to upon loss or damage of a leg is an amputation experiment, which reduces the effect of afferent information from sensory organs in insect legs. Behavioural studies have reported adaptive spatiotemporal coordination patterns after leg amputation in the cockroach^1^, the stick insect^2,3^, the desert ant^29,30^, and the fruit fly^31^. We focused on the changes in electromyographic (EMG) signals of the leg muscles responsible for locomotion before and after leg amputation. Such a focus, which has not been previously examined, is advantageous because: (1) EMG signals indirectly reflect the neural activities of thoracic ganglia and lower motor neurones involved in leg coordination; and (2) we can measure EMG signals during natural locomotion without disrupting the insect sensory-motor loop.

We aim to elucidate the adaptive inter-leg coordination mechanism of the crickets (*G. bimaculatus*), which respond to leg amputation patterns similarly to other insects. Simultaneous recording of leg movements, velocity in the walking direction, and angular velocity of body rotation showed invariant and variant patterns in remaining leg coordination before and after amputation. Simultaneous recordings of EMG signals and leg movements during walking both before and after leg amputation showed that the activation timing of levator muscles in both midleg muscles synchronised in phase when the legs were amputated at coxatrochanteral (CTr) joint, whereas the timing showed both anti-phase and in-phase synchronisation when only one leg or one leg and a femur remained. These results suggest that an intrinsic contralateral connection exists within the mesothoracic ganglion^21,32–34^, and mechanoreceptive informational feedback from the campaniform sensilla^26,35–37^ of the legs overrides the centrally generated patterns, resulting in the anti-phase leg movements of a normal gait.

## Results

### Walking gait pattern in the cricket

**Figure 1:**
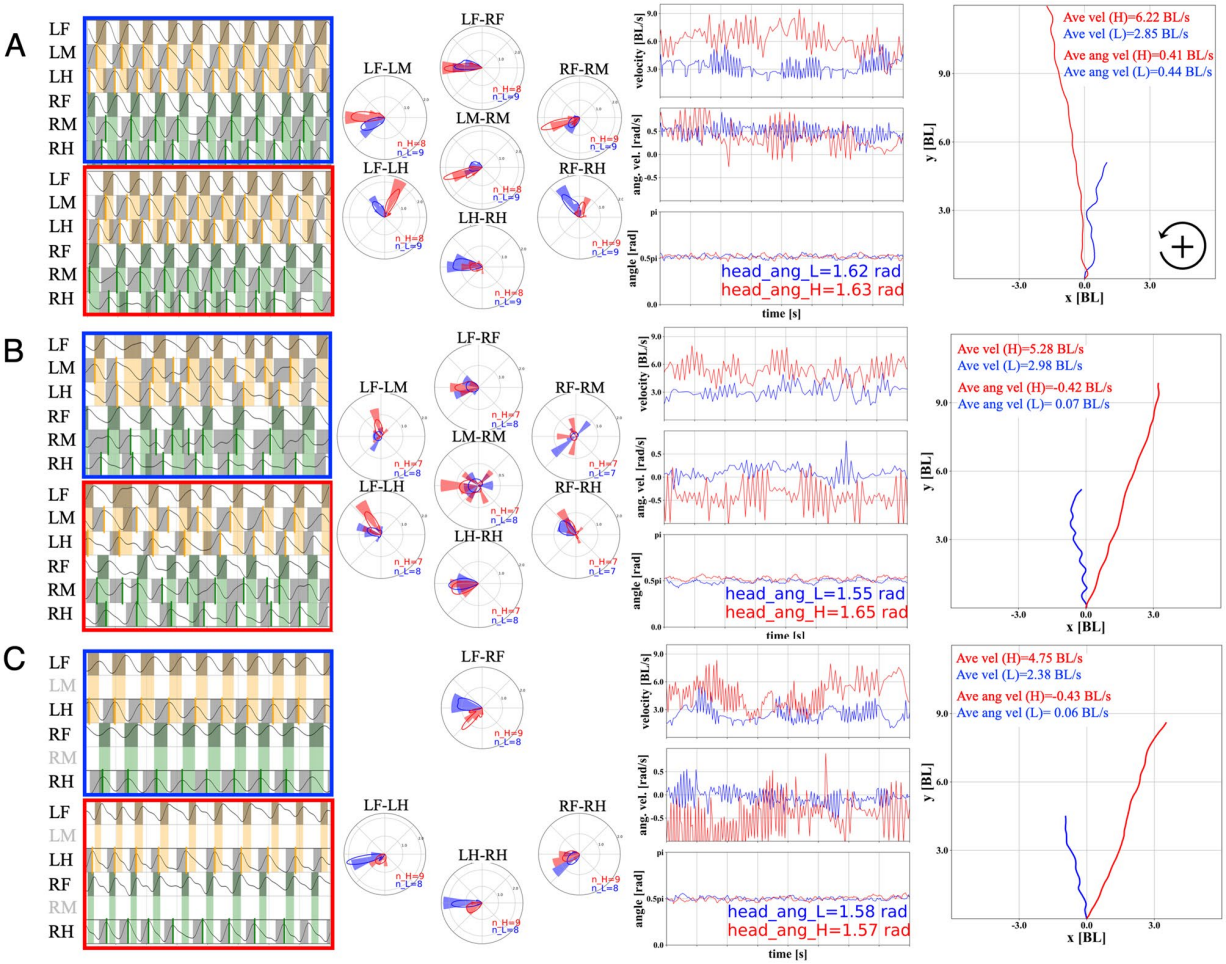
Gait pattern of the cricket (all data in 2 s intervals, blue and red colours represent gait data of low and high walking velocities, respectively). (**A**) Intact, blue = 2.85 BL(Body Length)/s, red = 6.22 BL/s; (**B**) Both middle legs amputated at the femur-tibial (FTi) joints, blue = 2.98 BL/s, red = 5.28 BL/s; and **C**. Both middle legs amputated at the coxatrochanteral joint (CTr) joints, blue = 2.38 BL/s, red = 4.75 BL/s. The far left panels show gait diagrams in walk, representative of each condition. The black lines show normalized leg joint trajectory of each leg estimated by DeepLabCut. The grey coloured region indicates swing phase (recovery stroke) periods, in which a leg moves forward. The orange and green coloured region indicate the swing phase of LF and RF legs. Bold lines (LM and LH: orange, RM and RH: green) on the gait diagrams show the timing of foot contact on legs for visibility. The second panels from the left show phase difference between the foot contact timings of legs using circular histogram and kernel density estimation (KDE) plots (left top: LF-LM, left bottom: LF-LH, centre top: LF-RF, centre middle: LM-RM, centre bottom: LH-RH, right top: RF-RM, right bottom: RF-RH). The third panels from the left show measured walking velocities [BL/s] in the walking direction (top), angular velocities [rad/s] of body rotation in the yaw axis (middle), and head angles $\theta _{head}^{cricket}$ [rad] (bottom) during walking. The far right panels show walking trajectories in the *x*-*y* plane [BL], which are integrated over the 2 s from the origin (0, 0) by using velocity, angular velocity, and body angle data for each condition.

We simultaneously measured leg movements, locomotion speed, and moving direction (body rotational velocity) during treadmill walking (See “Methods” and Supplemental Materials for details) for evaluation of the coordination pattern between legs before and after leg amputations. Figure 1 shows representative gait patterns for the conditions (A) intact walking, (B) both middle legs amputated at the FTi joints, and (C) both middle legs amputated at the CTr joints. In this section, we focus on the coordination between remaining legs, and we focus on the amputated leg’s muscle activation patterns in the next subsection.

**Figure 2:**
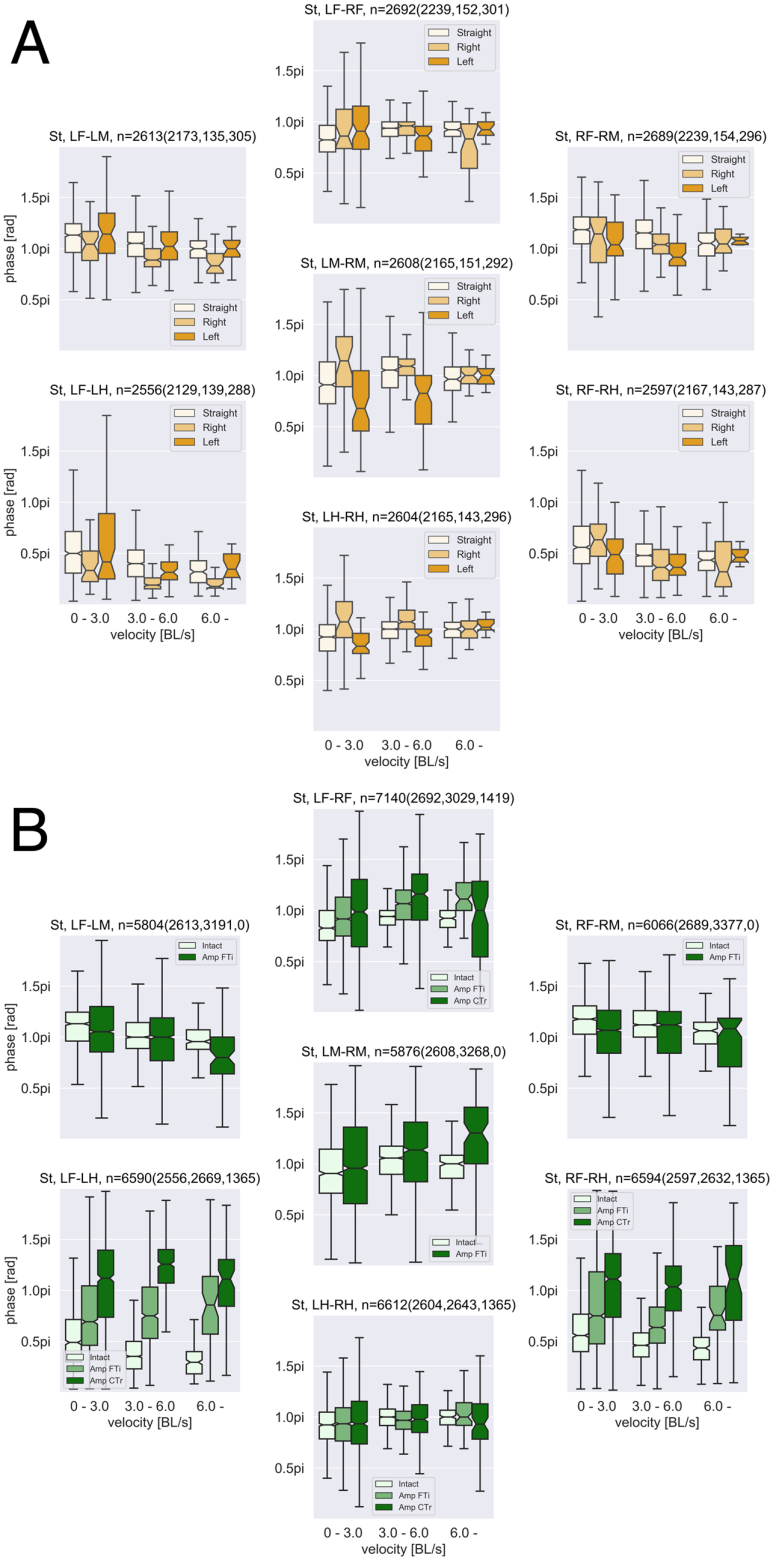
(**A**) Effect of body rotation on leg coordination. (**B**) Effect of leg amputation on leg coordination. Each panel on A and B show phase differences between foot contact timings (from swing to stance phases) of legs (left top: LF-LM, left bottom: LF-LH, centre top: LF-RF, centre middle: LM-RM, centre bottom: LH-RH, right top: RF-RM, right bottom: RF-RH). *n* indicates the analysed periods for each panel (**A**: n = All (Straight, Right, Left) and **B**: n = All (Intact, Amp FTi, Amp CTr)). In (**A**), the colour difference in each panel represents walking direction: Straight = − 1.0 to 1.0 rad/s, Left $> 1.0$ rad/s, Right $< -\,1.0$ rad/s. In (**B**), the colour difference represents the conditions of A, B, and C in Fig. 1. Horizontal axes represent walking velocity (from 0.0—3.0, 3.0–6.0, 6.0– BL/s).

To clearly define the starting point of this study, we first focused on the leg coordination during the intact walk in Fig. 1A. In the representative low speed walk (2.85 BL(Body Length)/s (blue) in Fig. 1A), the intact cricket exhibited a tetrapod gait, where phase differences between fore and hind legs were approximately 0.75 $\pi$. In contrast, in the high speed walk (6.22 BL/s (red) in Fig. 1A), the gait approached a tripod gait, but it was not a “strict” tripod gait because the phase differences between fore and hind legs stayed at approximately 0.25$\pi$, which was not in-phase with the typical tripod. We found similar trends in all intact walk data for more than 10,000 periods for all legs in six crickets (N = 6) (Fig. 2B). It should be noted here that phase differences between contralateral legs, i.e., LF (left fore)-RF (right fore), LM (left middle)-RM (right middle), and LH (left hind)-RH (right hind), show anti-phase synchronization, where phase differences were approximately $\pi$, independent of the locomotion speed.

During the experiments, we measured rotational angular velocities of the spherical treadmill in the three directions (yaw, roll, and pitch) by using two 2-D optical flow sensors attached to the treadmill base^31,38^ (see “Methods” section and the Supplemental Materials). We can calculate locomotion speed in the walking direction and body rotational angular velocity with the sensor values. Additionally, pose estimation algorithms with deep learning, i.e., DeepLabCut^39,40^, for measured two dimensional camera images during the walk facilitated the detection of the body and head angles of the cricket on the absolute coordinates as well as leg movements for the gait analysis. The third panels from the left in Fig 1 show the locomotion speed (top), rotational angular velocity (middle), and head angles (bottom) for each condition. The far right panels show the calculated trajectories of the cricket walk using the optical flow sensor values and estimated body angles (See “Methods” for details) for 2 s in each condition. The locomotion trajectories obtained indicated that the cricket walked almost in a straight direction in our experimental setup.

Figure 2A shows the statistical analysis of the effect of body rotation on gait patterns. Each panel shows a phase difference between the corresponding legs, for example, LF-RF, depending on the body rotation and locomotion speed. The colour difference in each panel represents direction of the walk: Straight = − 1.0 to 1.0 rad/s, Left > 1.0 rad/s, Right < − 1.0 rad/s. For the phase differences of the middle and hind legs (LM-RM and LH-RH), we found the effect of body rotation, meaning that the phase difference exceeded $\pi$ for right rotation (< − 1.0 rad/s), whereas the phase difference did not exceed $\pi$ for left rotation (> 1.0 rad/s). However, for the other phase relationships, we could not find any trends. Furthermore, phase differences for higher locomotion speeds were not significantly different depending on body rotation direction. The facts suggest that effect of turning was small on gait patterns in our experimental setups. We used all data including more than 1.0 rad/s and less than − 1.0 rad/s for the leg coordination and muscle activation analysis in this study (Fig. S9 show the results on more narrow range of angular velocity for the straight walk (− 0.5 to 0.5 rad/s), suggesting the independent of the body rotation).

The gait patterns of the crickets that underwent amputation varied depending on which legs had been removed. The movements of the remaining femur of the amputated middle legs were synchronized anti-phase and in-phase ((Fig. 1B; center circle (LM-RM phase difference) in the bottom panel). In contrast, LF-RF and LH-RH legs remained in anti-phase synchronization similar to the intact walking. Phase difference between fore and hind legs (LF-LH and RF-RH) was approximately 0.75$\pi$, like a tetrapod gait not depending on the locomotion speed. We found similar trends in leg coordination as shown in the statistical data of Fig. 2B (Fig. S10 shows the circular statistics). For crickets with amputations of the both middle legs at the CTr joints, the gait pattern was a so-called ‘trot’ gait, in which diagonal pairs of legs (RF-LH or LF-RH) contacted the ground almost simultaneously (Fig. 1C). Moreover, Fig. 2B (Fig. S10) statistically indicates the modified leg coordination pattern after leg amputation, with no significant effect of locomotion speed.

### Activation of leg muscles

We measured leg movements and EMGs under four conditions: (a) Intact walking; (b) Right middle leg amputated at the femur-tibia (FTi) joint; (c) Right middle leg amputated at the coxatrochantecral (CTr) joint; and (d) Both middle legs amputated at the CTr joint. Figure 4A (b), in which the right midleg is amputated at the femur-tibia (FTi) joint, shows a double-period stance phase in the amputated leg (b-2). Upon contact with the ground, the truncated midleg moved as the stance leg in the condition. However, as the shortened leg was unable to support the body, it immediately shifted to a swing phase, resulting in a reduced stance phase. The leg then touched the ground again, and cycled once more through a shortened stance phase, leading to a double period stance phase. For an amputated leg that was able to support the body upon contact with the ground, a period stance phase consistent with the tripod gait occurred, as shown in the first period on (b-2) of Fig. 4A. This fact preserves the consistency with the gait analysis on remaining legs.

Figure 4A shows the measured EMGs and gait diagrams for the four conditions of leg amputation (a) to (d). The lower graphs in Fig. 4A illustrates activation of the levators in the LM and RM legs and the retractor in RH leg with highlights during 4 periods. These graphs clearly show that the levator activation begins at the pre-swing phase while the retractor activation begins at the early stance phases. Spike train, which is defined as a list of the times where spikes have occurred, was generated by using a threshold for each EMG signal (0/1 for less/greater than the thresholds). Gait cycle period and frequency were calculated using the obtained gait diagrams. Power spectrum density analysis was conducted to detect the characteristic frequencies of raw EMGs and spike trains with respect to gait patterns. Figure 4B shows gait cycle period, frequency, and power spectrum density for raw EMGs and their spike trains in Fig. 4A(a). The graphs also indicate that peak EMG signal frequency occurs between 3 and 4 Hz, corresponding to gait cycle frequency.

**Figure 3:**
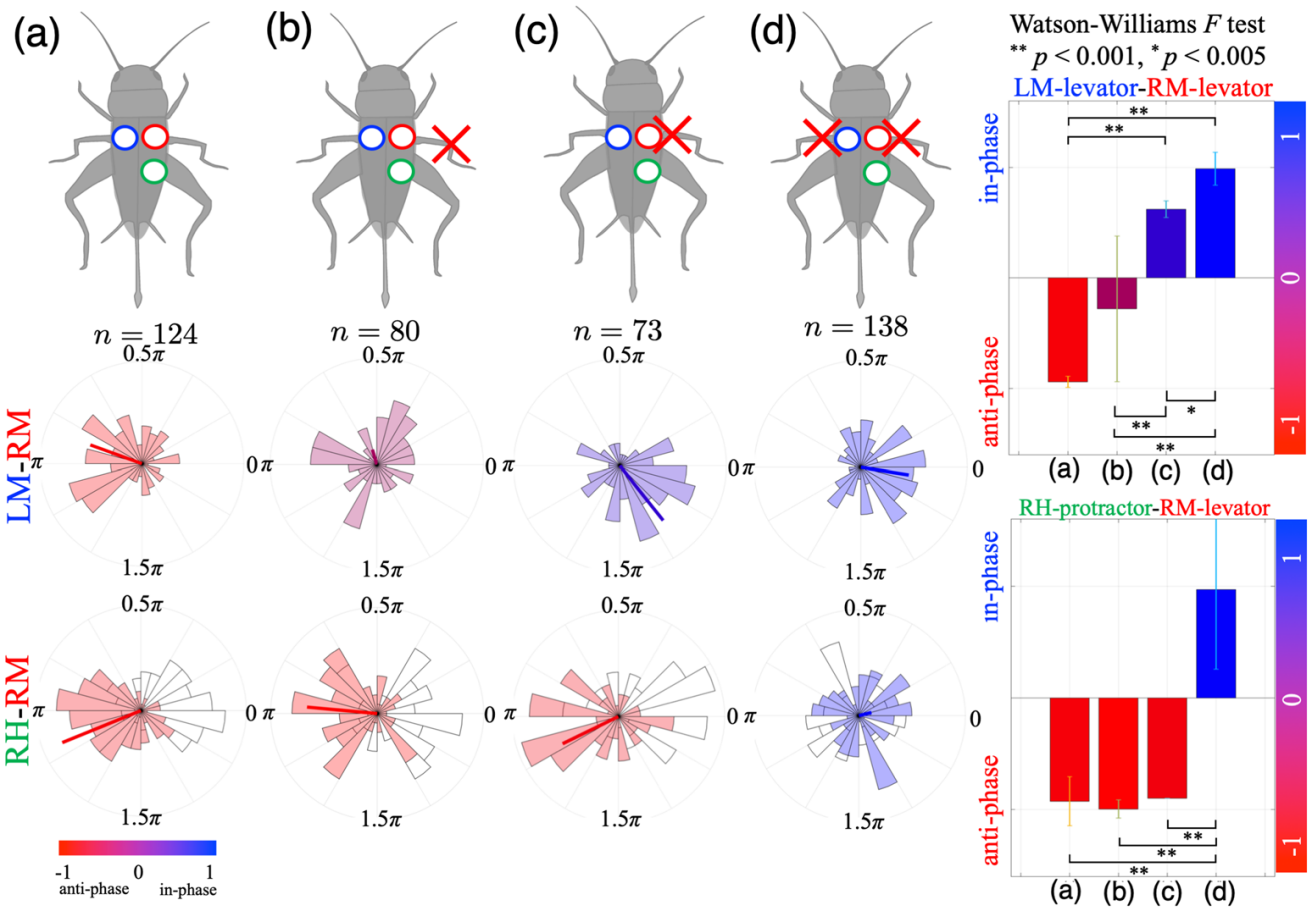
Change in muscle activation timing before and after leg amputations. The phase relationships of leg muscle activations between the LM and RM levators (left middle panels) and between the RM levator and RH protractor (left bottom panels) in conditions (**a**–**d**) using circular histogram plots. Experimental conditions: (**a**) Intact walking; (**b**) Right middle leg amputated at the FTi joint; (**c**) Right middle leg amputated at the CTr joint; (**d**) Both middle legs amputated at the CTr joint. We changed the experimental conditions in the order from (**a**) to (**d**) by amputating cricket legs at 3 different positions as shown in the upper left panels. Walking during more than 5 periods was analysed as one trial for each condition. To calculate the phase relationship of muscle activations in the corresponding trial, we conducted cross-spectrum analysis^41^. The bold line in the left panels shows average phase; the line length represents the standard deviation (0 to 1, with a larger deviation approaching 0 and a smaller deviation approaching 1) of the phase relationships of muscle activations over all trials. The colours (histogram and bar) are indices representative of degrees of in-phase/anti-phase synchronisation $I_{sync}$ (see “Methods” section). The right panels show changes in the average phase and standard deviations on the RM-LM and RM-RH muscles over the four conditions. The phase relationship between the LM and RM leg muscles gradually changes from anti-phase synchronisation in (**a**) to in-phase synchronisation in (**d**). For the RH and RM muscles, the phase relationship shifts to in-phase synchronisation in condition (**d**). The Waston-William *F* test was used to reveal differences in phase relationships between the conditions. A *p*-value of 0.005 was set as the criterion for statistical significance.

**Figure 4:**
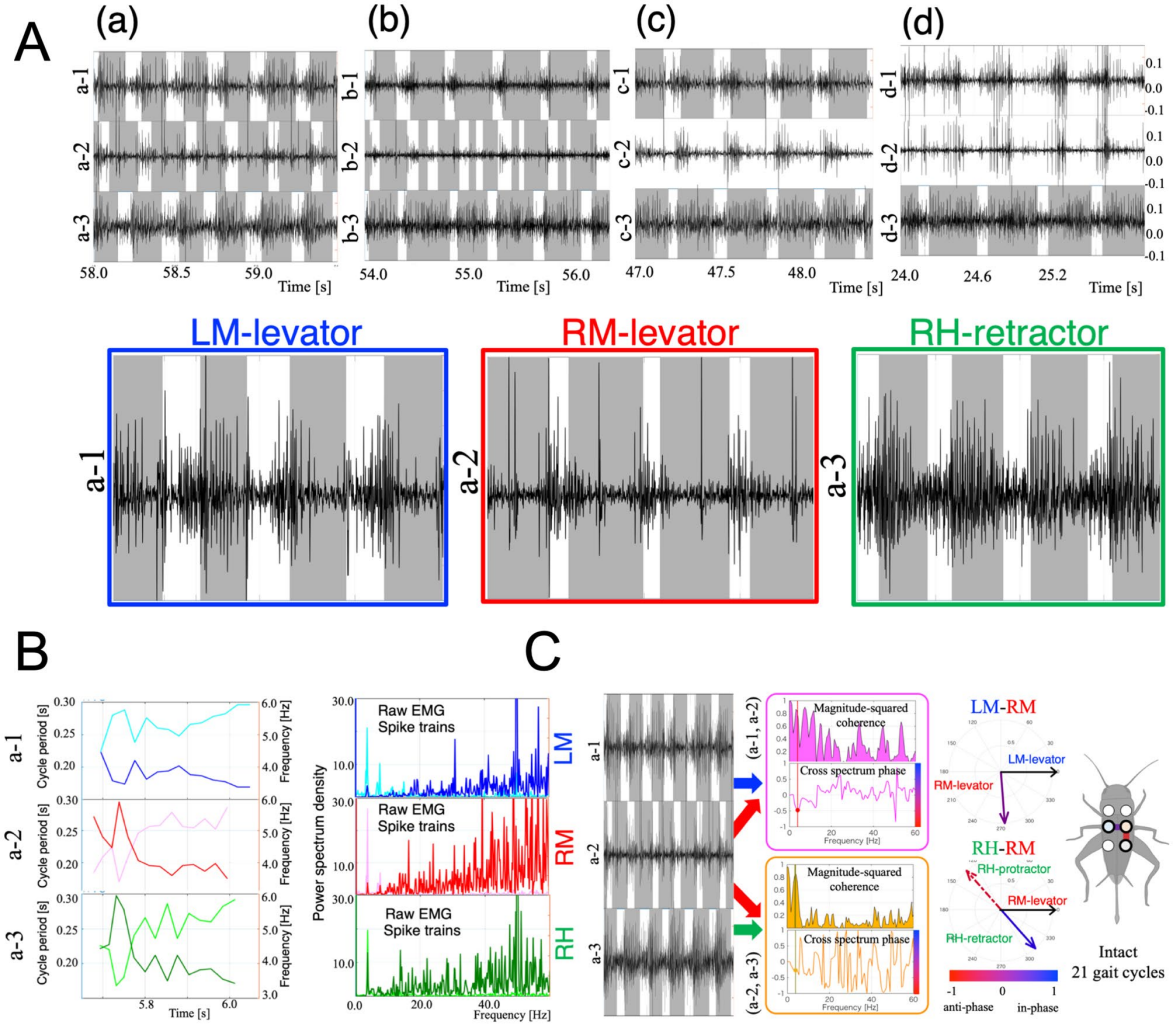
(**A**) Measured EMGs and gait diagrams for four conditions of leg amputation (a) to (d) on Setup 2. In each panel, the upper (e.g. a-1), middle (e.g. a-2), and lower (e.g. a-3) graphs shows the EMG and gait diagram in the left middle (LM, blue), right middle (RM, red), and right hind (RH, green) legs, respectively. The gait diagrams were drawn by visually measuring the duration of the stance phase, which is the period during which legs contact the treadmill. In the gait diagrams, the coloured regions represent the stance phases. The lower graphs illustrate activation of the levators in the LM and RM legs and the retractor in RH leg with highlights during 4 periods. (**B**) Gait cycle period, frequency, and power spectrum density for raw EMGs and their spike trains in **A**(a). The upper, middle, and lower graphs shows the data from the LM (a-1), RM (a-2), and RH (a-3) legs, respectively. The three graphs on the left show the gait cycle period (light blue, red, and green) and frequency (dark blue, red, and green) that we calculated using the gait diagrams. The three graphs on the right represent the power spectrum density of raw EMGs (dark blue, red, and green) and their spike trains (light blue, red, and green). (**C**) Cross-spectrum analysis for intact walking (**A**)(a) over 21 gait cycles. Cross-spectrum analysis extracts a common frequency component and its phase relationship (relative activation timing) between two EMG signals. The upper and lower graphs in the middle region show the results of the analysis for EMG signals of LM-RM (a-1 and a-2, magenta) and RM-RH (a-2 and a-3, yellow), respectively. The upper panels in each graph show magnitude-squared coherence, and the lower panels show the cross-spectrum phase for each frequency. The right circles indicate polar plots of the obtained phase relationship between RM-LM and RM-RH EMGs. The dotted lines in RM-RH polar plots show ’virtual’ protractor muscle activation timing in the RH leg by hypothesising ’quasi’ anti-phase activation against the retractor muscle.

To quantify changes in EMG patterns before and after leg amputation, we analysed changes in relative activation timing between muscles (LM-RM and RM-RH) in conditions (a)–(d). Walking during more than 5 periods was analysed as one trial for each condition. To calculate the phase relationship between muscle activities (e.g. the RM and LM levators) in the corresponding trial, we conducted cross-spectrum analysis^41^ (Fig. 4C, see “Methods” for details). Data obtained from cross-spectrum analysis are angles, thus, we applied circular statistics (see “Methods”). Figure 3 shows the phase relationship of leg muscle activation between the LM and RM levators (left middle panels) and between the RM levator and RH protractor (left bottom panels) in the conditions (a)–(d) using circular histogram plots. For each cricket (N = 5), we measured EMGs and leg movements in order from conditions (a) to (d) by amputating cricket legs at 3 different positions as shown in the left upper panels in Fig. 3. *n* indicates the trial number. The bold line in the left bottom panels shows average phase; the line length represents its standard deviation (0 to 1, with a larger deviation approaching 0 and a smaller deviation approaching 1) of the phase relationships of muscle activation over all trials. The colours are indices representative of degrees of in-phase/anti-phase synchronisation $I_{sync}$ (see “Methods” section). The right panels show changes in the average phase and standard deviations on the RM-LM and RM-RH muscles over the four conditions. The results indicate that the timing of muscle activities between the LM and RM are modulated from anti-phase to in-phase synchronisation throughout the course of the experimental conditions (a) to (d), whereas the timing between RM and RH significantly changes between the conditions (c) and (d).

## Discussion

We investigated changes in leg coordination patterns of the cricket (*G. bimaculatus*) using two experimental setups: (1) simultaneous recordings of leg movements, walking velocity and body rotation, and (2) simultaneous recordings of muscle activations and leg movements. The gait analysis for remaining leg coordination indicated that phase difference of the foot contact timing between fore and hind legs were changed from almost in-phase (0.25 $\pi$) to anti-phase ($\pi$) synchronization before and after amputations, resulting in the change from a tetrapod/tripod gait to a four legged trot gait. The EMG analysis indicated that amputation of the midlegs changes the coordination patterns of leg muscle activations according to the residual function of the amputated legs. Activation timing of the midleg muscles synchronised in phase when both legs were completely amputated, whereas the activation timing showed both anti-phase and in-phase synchronisation when one leg remained or one leg and a femur remained, which preserve the consistency with the gait analysis on remaining legs.

Previous studies have reported adaptive locomotion after leg amputation in other insects as well as crickets we here showed. For example, cockroaches exhibited adaptive modulation of leg tip trajectories after one- or two-leg amputation^1^. Altered gait patterns, dependent on which leg(s) were amputated, have also been reported in the stick insect, suggesting that afferent information from the legs affects coordination^2,3^. Studies on the desert ant which provide evidence of a step counter that measures distance travelled during navigation^29^ also suggest that gait patterns remain unchanged despite imposed alterations in leg length^30^. Berendes et al. (2016) reported speed-dependent movements in the remaining legs of the fruit fly (*Drosophila*) after a single-leg amputation^31^. Our results also suggested consistent insights with previous works, showing invariant and variant patterns in remaining leg coordination before and after leg amputation: phase differences between contralateral legs were invariant on anti-phase synchronization, whereas phase difference between ipsilateral legs, e.g., fore and hind legs was variant.

Speed and direction dependency of gait patterns are essential in defining the starting point of the discussion. In our experiments in Setup 1, the intact walking transition from a tetrapod gait to a tripod-like gait in which contralateral legs remain in anti-phase synchronization, whereas the phase difference between the fore and hind legs mainly shifts from 0.5 $\pi$ (tetrapod) to 0.25 $\pi$ (‘quasi’ tripod). The relatively small modification of gait patterns could be related to the narrow bandwidth (around 3–5 Hz in Fig. S8B) of walking frequency in cricket locomotion. Studies^13,15,42^ on gait modification that depend on speed have shown the variant walking period and the invariant swing phase (recovery stroke) period. Our gait analysis also indicated the same trends in the walking period and swing phase period (Fig. S8A–C). The movements of the remaining femur of the amputated middle legs (Fig. 1B) were synchronised in anti-phase and in-phase, and then, with the increase in locomotion speed, the average phase difference between the middle legs shifted to in-phase synchronization (Figs. 2B, S10), suggesting a strong connection in leg coordination in the high speed walk reported in^31^, who reported ipsilateral coordination. The contralateral coordination shown here provides new insights. Regarding the effect of turning direction on our experimental setup, we found that the rotational effect was not significant on gait pattern (Fig. 2A) due to the small curvature of the walking trajectories: the curvature was not turning^42^ *per se*.

Our experiments indicated that the muscle activation timing of the midlegs was synchronised in phase after both legs were completely amputated. Some crustaceans, including crayfish, lobster, and shrimp use swimmerets, which are paired appendages on each segment of abdomen, to swim and to ventilate eggs. The movement of swimmerets has been found to be a contralateral in-phase synchronised pattern^32^. Moreover, crickets placed on the surface of water^43,44^, exhibit a breast-stroke-like swimming gait, in which the left and right legs move in phase. Knebel et al. found that pro- and mesothoracic ganglia of the locust exhibit in-phase synchronisation patterns *in vitro* in the absence of any sensory information or descending commands^21^. These previous studies suggest the existence of intrinsic contralateral connections within ganglia that coordinate the in-phase contralateral movement patterns of the midlegs. Potential advantages of in-phase synchronisation between contralateral legs are: (1) energetic efficiency^34^, and (2) stability of the in-phase oscillating mode from the viewpoint of a dynamical system^45^.

Recent in vitro studies have reported the significant contribution of the subeshophageal ganglion (SEG) to leg coordination in insect locomotion. Headless insects with neither brain nor SEG do not exhibit spontaneous walking, except only briefly under tactile stimulation. In headless crickets, for example, defecation initiates walking, although the tripod walking pattern is never achieved^46^. In comparison, headless insects left with an intact SEG walk spontaneously absent any external stimulation. Knebel et al. investigated the functional connections between leg CPGs and the SEG in the locust^23^. They found that the SEG not only activates leg CPGs, but it also modulates all bilateral inter-CPG connections, resulting in the in-phase oscillation of the ganglia. The in-phase synchronisation of midleg muscle activation that we observed in in vivo amputation experiments also suggests a contribution of the SEG to in-phase coordinated walking patterns. Furthermore, the SEG modulates inter-leg sensory-motor interaction through a contralateral pathway^22^. The study by Knebel reported that insect leg sensory-motor loops consist of a contralateral pathway to the head and an ipsilateral descending pathway, in which the SEG plays a crucial role in processing and transforming sensory information.

Sensory feedback plays an essential role in leg coordination during insect locomotion by modulating the timing and magnitude of centrally-generated patterns^22,26,34^. The exteroceptive and proprioceptive mechanoreceptors that cover the insect body, including the legs^47^ respectively comprise stretch receptors, e.g. chordotonal organs^18^, and the trichoid sensilla (hair plate located near the joints^48^) along with the campaniform sensilla (load receptors^35–37^). In the legs, these receptors contribute the following two sensory mechanism: (1) loading and unloading of the legs, and (2) leg movements^26^. The leg amputation experiments, which silence the campaniform sensilla by eliminating contact between the legs and the ground, indicate the significant contribution of campaniform sensilla to leg movement patterns; the receptors on the coxa and trochanter monitor the magnitude of the load on the legs^35–37^. The desert ant exhibits a robust walking gait despite changes in the leg length^30^, suggesting the contribution of the remaining campaniform sensilla of the coxa and trochanter. The four conditions of our experiments represent a spectrum of reduced effects of the campaniform sensilla on leg coordination, with anti-phase synchronisation reflecting the greatest effect, and in-phase synchronisation reflecting the least. This suggests that feedback from the mechanoreceptive campaniform sensilla overrides the centrally-generated coordination patterns of leg CPGs and the SEG, leading to the normal walking gait of intact insects. This finding is consistent with our previous studies pertaining to robotics^49,50^, in which we proposed a load-dependent leg coordination rule that exploits load sensory information from each leg.

The reason why we chose middle leg amputations in this study was that the middle legs play an essential role as an “ipsilateral mediator” by mainly supporting the body in hexapedal leg coordination: the fore legs have a role in probing (sensing environments)^25,42,51^ and the hind legs have a role in propulsion of the body for locomotion^51^, thus amputations of these legs would have a more significant impact on leg coordination for other functions, like sensing or generating forces for locomotion. Therefore, as a first step in the discussion of adaptive mechanisms, we consider that middle leg amputation was suitable for effective extraction of the underlying mechanism. Furthermore, mounting direction of the hind legs are opposite to the fore legs. Additionally, from the viewpoint of EMG measurement, leg segments in the fore legs are relatively small, making measurements increasingly difficult to perform, hence the second reason for middle leg amputation . Investigation of the fore and hind legs is quite interesting and meaningful for the complete understanding of the leg coordination mechanism, and we consider them imperative in future research.

The main contribution of our study is to show that the change in *in vivo* EMG patterns before and after leg amputation indicates the change from anti-phase to in-phase synchronisation based on the residual function of the amputated legs. This suggests a significant contribution on the part of the mechanoreceptive campaniform sensilla to leg coordination. A limitation of our study was that we did not measure neural activities, e.g., interneurones that participate in CPGs or motoneurones, during locomotion. Instead, we analysed only muscle activation patterns and the movements of legs before and after leg amputation. One potential future direction would entail additional experiments using a prosthetic leg corresponding to the amputated leg, as in previous studies^29,30,52^. A study that models leg CPGs, the SEG, and the sensory feedback mechanism would be useful for designing a decentralised controller for an insect-like six-legged robot.

## Methods

### Animals

The crickets (*Gryllus bimaculatus*) were raised in a laboratory colony. They were reared on a 14h:10h light and dark cycle at 28 ± 2 °C (lights on at 6:00). They were fed a diet of insect food pellets (Oriental Yeast Co., Tokyo, Japan) and water ad libitum. Adult crickets that had molted within the previous 2 weeks were used in this study.

### Behavior experiments

The crickets used were randomly selected from our colony. Each cricket was anaesthetised using CO_2_ gas before it was placed on a treadmill to observe its walking gait pattern. The handmade treadmill was composed of a Styrofoam sphere ($\phi$ 100 mm) that hovers above a stream of air flowing beneath it. The tip of a plastic rod ($\phi$ 2 mm) that was printed using a 3D printer (FORTUS250mc, Stratasys Ltd., Eden Prairie, MN, USA) was attached to the cricket thorax using an insect pressure-sensitive adhesive (Cockroach trap, Earth Corporation, Hyogo, Japan). The rod was attached to a ring that was fixed on a manipulator, by which the cricket was placed on the exact desired position of the Styrofoam sphere. A cricket on the treadmill was able to walk and change its orientation and ground clearance freely.

After the walking gait pattern of the intact cricket was recorded as a control, it was removed from the treadmill for surgical treatment. The cricket was anaesthetised again using CO_2_ gas, and then a leg was amputated using fine scissors. To investigate how leg amputation modifies the walking gait pattern, we amputated cricket legs at two different positions (for Setup 1): the both middle legs at the FTi joints; and the both middle legs at the CTr joints and at three different positions (for Setup 2): the right middle leg at the FTi joint; the right middle leg at the CTr joint; and both middle legs at the CTr joint. After leg amputation, the cricket was placed on the treadmill again to observe the walking gait pattern.

### Setup 1: Simultaneous recording of walking pattern and velocity

As the first step in the experiments, it was necessary to define the starting point of the discussion on gait modification before and after leg amputation. We quantified the relationship between leg coordination patterns, locomotion velocity, and body rotation before and after leg amputations. To this end, we used a high-speed camera (GC-P100, JVCKENWOOD Corporation, 120 fps) and two-dimensional optical flow sensors (PMW3901, Pimoroni, 50 Hz) (Supplementary Video S1). The cricket motion data (movies) were stored on an SD memory card in the camera. We measured rotational angular velocities of the spherical treadmill in three directions (yaw, roll, and pitch) using two optical flow sensors attached to the treadmill base^31,38^. The sensor values were quantified and stored by Raspberry Pi 3B+ (Raspberry Pi Foundation) via the SPI (Serial Peripheral Interface) communication protocol. For synchronization of the recorded movies and measured optical flow values, we used small LEDs (light emitting diodes). By blinking the LEDs with tactile switches and storing the time sequence data with the Raspberry Pi, we could synchronize motion images and sensor values for later gait analysis (see Fig. S2 in the Supplementary Materials).

### Setup 2: Simultaneous recording of electromyogram and walking pattern

Electrophysiological experiments were performed in order to record changes in the EMGs of leg muscles before and after leg amputation. We used a pair of enamel-coated copper wires ($\phi 50$ µm) as electrodes. The tips of the wire electrodes were implanted into the coxal levator muscles of the right and left middle legs and the retractor muscles of the right hind legs. The retractor muscles were chosen due to the difficulty in getting EMG data from protractor muscles due to muscle arrangement in the cricket (Fig. S1 in the Supplementary Materials). To implant the wire electrode, two fine holes were made in the cuticle using a sharpened insect pin. The tip of the wire electrodes were inserted through the hole into the target muscles and the wires were fixed on the surface of the cuticle with glue (Aron Alpha Jelly-like, Konishi, Japan). The electrodes were connected with amplifiers (DAM80, World Precision Instruments), and the signals of the EMGs were recorded using a data logger (GL900, GRAPHTEC, 5000 Hz) for later analysis. The walking patterns of the crickets were recorded using a high-speed camera (HAS-L1, DITECT, Japan, 640 × 480 pixels, 500 fps), and the images were saved as sequential tiff files on Windows PC for later analysis. For simultaneous recording of EMGs and leg movements, we used the high-speed camera (HAS-L1) and the data logger (GL900), which was controlled by software (DIPP-ADII, DITECT, Japan) that enabled us to synchronise EMG signals with a high-speed movie of the walking cricket (Supplementary Video S2). To analyse gait patterns of the crickets, gait diagrams were drawn by visually measuring the duration of the stance phase, i.e. the period when the legs are in contact with the treadmill.

### Gait data analysis

To quantify leg coordination patterns for the measured 2-D high speed camera images (640 × 320 pixels at 120 fps), we used a pose estimation algorithm with deep learning, i.e. DeepLabCut^39,40^. By marking training data(50–200 frames for each condition) for pose estimation, the algorithm automatically estimate 21 positions (Head, Pro, Meso, Meta, LF1, LF2, LM1, LM2, LH1, LH2, RF1, RF2, RM1, RM2, RH1, RH2, Bar, Axis, Fix, LED1, and LED2) during one trial (Fig. S2 and Supplementary Video S3). The average of the estimated position error for test data on each condition was 1.643 ± 0.345 pixels. For the estimated position data, we numbered the leg movements during the power and recovery strokes (stance and swing phases), leading to the quantification of the gait diagram. Phase differences between legs, e.g., LF-RF, were calculated by using the following equation:

1 $$\begin{aligned} \phi _{i}^{LF-RF} = 2\pi \dfrac{t_{TD(i)_{RF}} - t_{TD(i)_{LF}} }{T_{period(i)_{LF}}} = 2\pi \dfrac{t_{TD(i)_{RF}} - t_{TD(i)_{LF}} }{t_{TD(i+1)_{LF}} - t_{TD(i)_{LF}}}, \end{aligned}$$

where $T_{period(i)_{LF}}$ represents time duration of (*i*)th LF leg period and $t_{TD(i)_{RF}}$ represents touch down (TD) timing of the RF leg during (*i*)th period. By using the cricket body positions (Head, Pro, Meso, Meta) and the frame positions of the treadmill base (Bar, Axis, and Fix), head and body angles ($\theta _{head}^{cricket}$ and $\theta _{body}^{cam}$) were calculated during walking as shown in Fig. S2 on the Supplementary Materials. Blinking LED positions on camera images (LED2) were used for the synchronization with optical flow sensor values (Fig. S3).

By using two 2-D optical flow sensor values ($dx_1, dy_1, dx_2, dy_2$), the rotational angular velocities of the spherical treadmill in the yaw, roll, and pitch directions were calculated using the following equations^38^:

2 $$\begin{aligned} \omega _{yaw}= & {} - ( \sigma _{x_1} dx_1 + \sigma _{x_2} dx_2) \cos 45^{\circ }, \end{aligned}$$

3 $$\begin{aligned} \omega _{roll}= & {} \dfrac{1}{2} (- \sigma _{y_1} dy_1 + \sigma _{y_2} dy_2), \end{aligned}$$

4 $$\begin{aligned} \omega _{pitch}= & {} ( \sigma _{x_1} dx_1 - \sigma _{x_2} dx_2) \sin 45^{\circ }, \end{aligned}$$

where $\sigma _{x_1}, \sigma _{y_1}, \sigma _{x_2}, \sigma _{y_2}$ denote the scale parameters for changing millimetre length scale [mm/s] to radian angle scale [rad/s] (see Fig. S4 and Supplementary Materials for details). Then, according to the definition of the coordinate on the camera frame, the locomotion velocities in the *x* and *y* direction on the frame were calculated using the sphere radius ($l_s=50$ mm) as follows:

5 $$\begin{aligned} {\dot{x}}^{cam}= & {} l_s \omega _{roll}, \end{aligned}$$

6 $$\begin{aligned} {\dot{y}}^{cam}= & {} l_s \omega _{pitch}, \end{aligned}$$

7 $$\begin{aligned} v^{cam}= & {} \sqrt{({\dot{x}}^{cam})^2 + ({\dot{y}}^{cam})^2} = l_s \sqrt{\omega _{roll}^2 + \omega _{pitch}^2}. \end{aligned}$$

To normalize the length and velocity data depending on the body length of crickets, we divided the length and velocity data by using the corresponding cricket’s body length (BL) in Table S1, resulting in the unit transformation from mm or mm/s to BL or BL/s.

The cricket’s walking trajectories were integrated on the cricket frame coordinate by using the velocities in Eqs. (5) and (6) with cricket body angle $\theta _{body}^{cam}$ on the camera frame coordinate by using the following equations:

8 $$\begin{aligned} x^{cricket}(t+\varDelta t)= & {} x^{cricket}(t) + \varDelta t \{ {\dot{x}}^{cam}(t) \sin \theta _{body}^{cam}(t) - {\dot{y}}^{cam}(t) \cos \theta _{body}^{cam}(t) \}, \end{aligned}$$

9 $$\begin{aligned} y^{cricket}(t+\varDelta t)= & {} y^{cricket}(t) + \varDelta t \{ {\dot{x}}^{cam}(t) \cos \theta _{body}^{cam}(t) + {\dot{y}}^{cam}(t) \sin \theta _{body}^{cam}(t) \}, \end{aligned}$$

where initial position was set on the origin, i.e., $x^{cricket}(0), y^{cricket}(0) = (0,0)$, for each trial.

### EMG data analysis

To quantify changes in EMG patterns before and after leg amputation, we analysed changes in relative activation timing between muscles (LM-RM and RM-RH) in conditions (a)-(d). Accordingly, cross-spectrum analysis^41^ was conducted in order to detect common frequency components and their phase relationship (relative activation timing) between two EMG signals. By using this analysis, we were able to detect changes in the relative muscle activation timings between the LM and RM, or the RM and RH EMG signals and observe their effects on gait cycle frequency (3-4 Hz) before and after leg amputation (Fig. 4C). We selected a frequency for the cross-spectrum analysis based on the power spectrum density of each EMG signal. The peak frequency around the appropriate walking period was chosen for the analysis.

Data obtained from cross-spectrum analysis are angles, which represent the phase relationship between the activation timings of two corresponding EMGs. Thus, we applied circular statistics for statistical analysis using a circular statistics toolbox^53^ for MATLAB (Mathworks Inc., Natick, MA, USA) in order to calculate means and standard deviations of the relative phase relationship of two EMGs (LM-RM/RM-RH) for each condition. The colours in Fig. 3 represent the degree of in-phase/anti-phase synchronisation index $I_{sync}$: red indicates the anti-phase ($I_{sync}=-1$) and blue indicates the in-phase synchronisation ($I_{sync}=1$), which is calculated by the following equation:

10 $$\begin{aligned} I_{sync}=\cos {\hat{\theta }} \end{aligned}$$

where ${\hat{\theta }}$ represents the average phase. The Waston-William *F* test was used to reveal differences in phase relationships between the conditions. A *p*-value of 0.005 was set as the criterion for statistical significance.

## Supplementary Information

Supplementary Information 1.

Supplementary Video S1.

Supplementary Video S2.

Supplementary Video S3.

Supplementary Video S4.

Supplementary Video S5.

Supplementary Video S6.

Supplementary Video S7.

Supplementary Video S8.

Supplementary Video S9.

## References

[1] Hughes G. M (1957). The co-ordination of insect movements ii. the effect of limb amputation and the cutting of commissures in the cockroach (*blatta orientalis*). J. Exp. Biol.

[2] Graham D (1977). The effect of amputation and leg restraint on the free walking coordination of the stick insect carausisus morosus. J. Comp. Physiol..

[3] Grabowska M, Godlewska E, Schmidt J, Daun-Gruhn S (2012). Quadrupedal gaits in hexapod animals – inter-leg coordination in free-walking adult stick insects. J. Exp. Biol..

[4] Popov AV, Shuvalov VF (1977). Phonotactic behavior of crickets. J. Comp. Physiol..

[5] Weber T, Thorson J, Huber F (1981). Auditory behavior of the cricket: I. dynamics of compensated walking and discrimination paradigms on the kramer treadmill. J. Comp. Physiol.

[6] Stout J, DeHaan C, McGhee R (1983). Attractiveness of the male acheta domesticus calling song to females. i. dependence on each of the calling song features. J. Comp. Physiol.

[7] Alexander RD (1961). Aggressiveness, territoriality, and sexual behavior in field crickets (orthoptera: Gryllidae). Behaviour.

[8] Hofmann HA, Stevenson PA (2000). Flight restores fight in crickets. Nature.

[9] Sakura M, Aonuma H (2013). Aggressive behavior in the antennectomized male cricket gryllus bimaculatus. J. Exp. Biol..

[10] Matsumoto Y, Mizunami M (2000). Olfactory learning in the cricket gryllus bimaculatus. Journal of Experimental Biology.

[11] Matsumoto Y, Mizunami M (2002). Temporal determinants of long-term retention of olfactory memory in the cricket gryllus bimaculatus. Journal of Experimental Biology.

[12] Matsumoto Y, Unoki S, Aonuma H, Mizunami M (2006). Critical role of nitric oxide-cgmp cascade in the formation of camp-dependent long-term memory. Learn Mem..

[13] Wilson DM (1966). Insect walking. Annu. Rev. Entomol..

[14] Graham D (1972). A behavioural analysis of the temporal organisation of walking movements in the 1st instar and adult stick insect ( carausius morosus). J. Comp. Physiol. A.

[15] Wosnitza, A., Bockemühl, T., Dübbert, M. H., Scholz & Büschges, A. Inter-leg coordination in the control of walking speed in *drosophila*. *J. Exp. Biol.* **216**, 480–491 (2013). 10.1242/jeb.078139 23038731

[16] Pearson KG, Iles JF (1969). Discharge patterns of coxal levator and depressor motoneurons of the cockroach, perillaneta americana. J. Exp. Biol..

[17] Pearson KG, Iles JF (1973). Nervous mechanisms underlying intersegmental co-ordination of leg movements during walking in the cockroach. J. Exp. Biol..

[18] Bässler U, Wegner U (1983). Motor output of the denervated thoracic ventral nerve cord in the stick insect carausius morsus. J. Exp. Biol..

[19] Dean J (1989). Leg coordination in the stick insect carausis morosus: effect of cutting thoracic connectives. J. Exp. Biol..

[20] Brekowitz A, Laurent G (1996). Central generation of grooming motor patterns and interlimb coordination in locusts. J. Neurosci..

[21] Knebel, D., Ayali, A., Pfüger, H. J. & Rilich, J. Rigidity and flexibility: the central basis of inter-leg coordination in the locust. *Front. Neural Circuits* **10**, 10.3389/fncir.2016.00112 (2017). 10.3389/fncir.2016.00112 PMC5225121 28123358

[22] Knebel D (2018). The subesophageal ganglion modulates locust inter-leg sensory-motor interactions via contralateral pathways. J. Insect Physiol..

[23] Knebel D, Rillich J, Nadler L, Pflüger HJ, Ayali A (2019). The functional connectivity between the locust leg pattern generators and the subesophageal ganglion higher motor center. Neurosci. Letters.

[24] Borgmann A, Hooper SL, Büschges A (2009). Sensory feedback induced by front-leg stepping entrains the activity of central pattern generators in caudal segments of the stick insect walking system. J. Neurosci.

[25] Berg EM, Hooper SL, Schmidt J, Büschges A (2015). A leg-local neural mechanism mediates the decision to search in stick insects. Current Biology.

[26] Ayali A (2015). Sensory feedback in cockroach locomotion: Current knowledge and open questions. J. Comp. Physiol. A.

[27] Menzel R, Giurfa M (2001). Cognitive architecture of a mini-brain: the honeybee. TRENDS in Cognitive Sciences.

[28] Alivisatos AP (2012). The brain activity map project and the challenge of functional connectomics. Neuron.

[29] Wittlinger M, Wehner R, Harald Wolf H (2006). The ant odometer: Stepping on stilts and stumps. Science.

[30] Wittlinger M, Wehner R, Harald Wolf H (2007). The desert ant odometer: a stride integrator that accounts for stride length and walking speed. J. Exp. Biol.

[31] Berendes V, Zill SN, Büschges A, Bockemühl T (2016). Speed-dependent interplay between local pattern-generating activity and sensory signals during walking in drosophila. J. Exp. Biol..

[32] Murchison D, Chrachri A, Mulloney BA (1993). Separate local pattern-generating circuit controls the movements of each swimmeret in crayfish. J. Neurophysiol..

[33] Büschges A, Schmits J, Bässler U (1995). Rhythmic pattern in the thoracic nerve cord of the stick insect induced by pilocarpine. J. Exp. Biol..

[34] Büschges A, Scholz H, El Manira E (2011). New moves in motor control. Curr. Biol..

[35] Zill SN, Keller BR, Duke ER (2009). Sensory signals of unloading in one leg follow stance onset in another leg: Transfer of load and emergent coordination in cockroach walking. J. Neurophysiol..

[36] Strauß, J. & Stritih, N. The accessory organ, a scolopidial sensory organ, in the cave cricket *troglophilus neglects* (orthoptera: Ensifera: Rhaphidophoridae). *Acta Zool. Stockholm)* **97**, 187–195 (2016).

[37] Dallmann CJ, Hoinville T, Dürr V, Schmitz J (2017). A load-based mechanism for inter-leg coordination in insects. Proc. R. Soc. B.

[38] Seelig J (2010). Two-photon calcium imaging from head-fixed drosophila during optomotor walking behavior. Nat Methods.

[39] Mathis A (2018). Deeplabcut: markerless pose estimation of user-defined body parts with deep learning. Nat Neurosci.

[40] Nath T (2019). Using deeplabcut for 3d markerless pose estimation across species and behaviors. Nat Protoc.

[41] Miller WL, Sigvardt KA (1998). Spectral analysis of oscillatory neural circuits. J. Neurosci. Method.

[42] Dürr, V., Theunissen, L., Dallmann, C., Hoinville, T. & Schmitz, J. Motor flexibility in insects: adaptive coordination of limbs in locomotion and near-range exploration. *Behav. Ecol. Sociobiol.*10.1007/s00265-017-2412-3 (2018).

[43] Kanou M, Morita S, Matsuura T, Yamaguchi T (2007). Analysis of behavioral selection after sensory deprivation of legs in the cricket gryllus bimaculatus. Zoological Science.

[44] Aonuma, H. *et al.* Cricket switches locomotion patterns from walking to swimming by evaluating reaction forces from the environment. In *AMAM2015: Proceedings of The 8th International Symposium on Adaptive Motion of Animals and Machines* (2015).

[45] Reches E, Knebel D, Rillich J, Ayali A, Barzel B (2019). The metastability of the double-tripod gait in locust locomotion. iScience.

[46] Naniwa K, Sugimoto Y, Osuka K, Aonuma H (2019). Defecation initiates walking in the cricket gryllus bimaculatus. J. Insect Physiol..

[47] Guthrie DM, Tindall AR (1968). The biology of the cockroach.

[48] Markle H (1962). Borstenfelder an den gelenken als schweresinnesorgane bei ameisen und anderen hymenopteren. Z. Vergl. Physiol..

[49] Owaki, D. & Ishiguro, A. A quadruped robot exhibiting spontaneous gait transitions from walking to trotting to galloping. *Sci. Rep.*10.1038/s41598-017-00348-9 (2017). 10.1038/s41598-017-00348-9 PMC5428244 28325917

[50] Owaki, D., Goda, M., Miyazawa, S. & Ishiguro, A. A minimal model describing hexapedal interlimb coordination: The tegotae-based approach. *Front. Neurorobot.*.10.3389/fnbot.2017.00029 (2017). 10.3389/fnbot.2017.00029 PMC5465294 28649197

[51] Dallmann, C. & Schmitz, J. Joint torques in a freely walking insect reveal distinct functions of leg joints in propulsion and posture control. *Proc. R. Soc. B* **283**, (2016). 10.1098/rspb.2015.1708 10.1098/rspb.2015.1708 PMC4795010 26791608

[52] Noah, J. *et al.* Walking on a ’peg leg’: Extensor muscle activities and sensory feedback after distal leg denervation in cockroaches. *J. Comp. Physiol.* **190**, 217–231 (2004). 10.1007/s00359-003-0488-x 14727135

[53] Berens P (2009). Circstat: a matlab toolbox for circular statistics. J. Stat. Softw.

