# Supplementary material for: Leg amputation modifies coordinated activation of the middle leg muscles in the cricket Gryllus bimaculatus
Source: Sci Rep. 2021 Jan 14;11:1327. doi: 10.1038/s41598-020-79319-6 (PMC7809211; doi:10.1038/s41598-020-79319-6)
Supplement: Supplementary file 1 — Supplementary Information 1. [file 41598_2020_79319_MOESM1_ESM.pdf]

Leg amputation modifies coordinated activation of the middle leg muscles in the cricket *Gryllus bimaculatus*

Dai Owaki[1,*], Hitoshi Aonuma[2], Yasuhiro Sugimoto[3], and Akio Ishiguro[4]

[1]Dept. of Robotics, Graduate School of Engineering, Tohoku University, Sendai ,980-8579, Japan

[2]Research Institute for Electronic Science, Hokkaido University, Sapporo, 060-0812, Japan

[3]Dept. of Mechanical Engineering, Osaka University, Suita, 565-0871, Japan

[4]Research Institute of Electrical Communication, Tohoku University, Sendai, 980-8577, Japan

*owaki@tohoku.ac.jp

Supplementary Video S1.

Setup1: Simultaneous recording of walking pattern and velocity in the cricket *Gryllus bimaculatus*

Supplementary Video S2.

Setup2: Simultaneous recording of electromyogram (EMG) and walking pattern in the cricket *Gryllus bimaculatus*

Supplementary Video S3.

Pose estimation with DeepLabCut (DLC) in the intact walking.

Supplementary Video S4 and S5.

Walking motion in the Fig.1 A (Intact walking) in 2s intervals: gait of low (S4) and high (S5) walking velocity.

Supplementary Video S6 and S7.

Walking motion in the Fig.1 B (Both middle legs amputated at the femur-tibial (FTi) joints) in 2s intervals: gait of low (S6) and high (S7) walking velocity.

Supplementary Video S8 and S9.

Walking motion in the Fig.1 C (Both middle legs amputated at the coxatrochanteral joints) in 2s intervals: gait of low (S8) and high (S9) walking velocity.

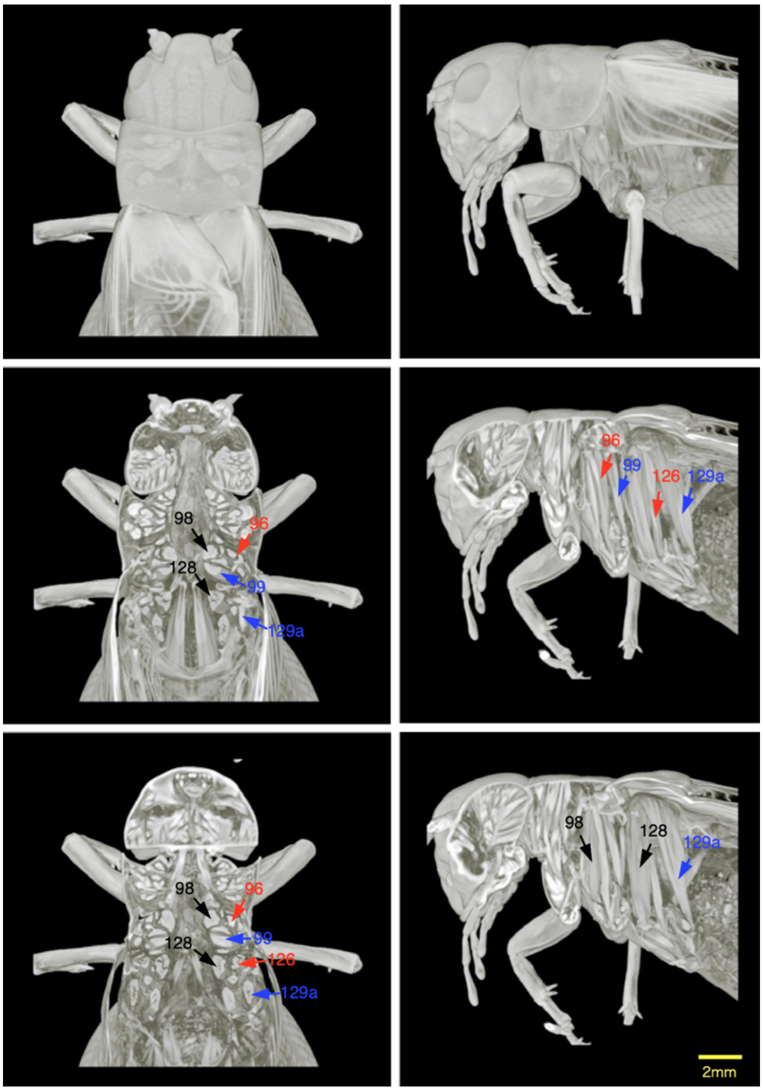

Fig.S1: X-ray micro-computed tomography (microCT) imaging. The crickets were anesthetized and fixed with alcoholic Bouin's fixative over night at room temperature. After fixation, they were dehydrated with ethanol series (70%, 80%, 90% and 100%). Then they were stained using 1% iodine in ethanol overnight in order to enhance contrast of each tissue when they were scanned by X-ray micro CT. After samples were rinsed with 100% ethanol, they were transferred to liquidized t-butyl alcohol series (50%, 75% and 100%). The samples were freeze-dried by using a vacuum evaporator (PX-52, Yamato Ltd., Japan) with a cold alcohol trap (H2SO5, AS ONE, Japan). All chemicals were obtained from Kanto Chemical Co. (Tokyo, Japan). Samples were scanned on an X-ray microCT system (inspeXio, SMX-100CT, Shimadzu Corporation, Kyoto, Japan) whose X-ray source was operated at 75kV and 40μA. The images were reconstructed with a voxel size of 5-20μm. Image reconstruction and rendering were carried out using VGStudio MAX (version 3.0, Volume Graphics, Heidelberg, Germany, https://www.volumegraphics.com/en/products/vgstudio-max.html). As reported in[S1], 98 and 128 (black) are the protractor muscles for the middle and hind legs, respectively. 99 and 129a (blue) are the retractor muscle for the middle and hind legs. 96 and 126 (red) are the levator muscle for the middle and hind legs. On the middle leg, the levator muscle 96 in the three kinds of muscles was the easiest to measure EMG signal because it located in the coxa whereas the others (98 and 99) located inside of the body. On the hind leg, the retractor muscle 129a was the easiest to measure EMG signal because it was located just under the cuticle, whereas the others (128 and 126) also located inside of the body, hence not easy to access these muscles.

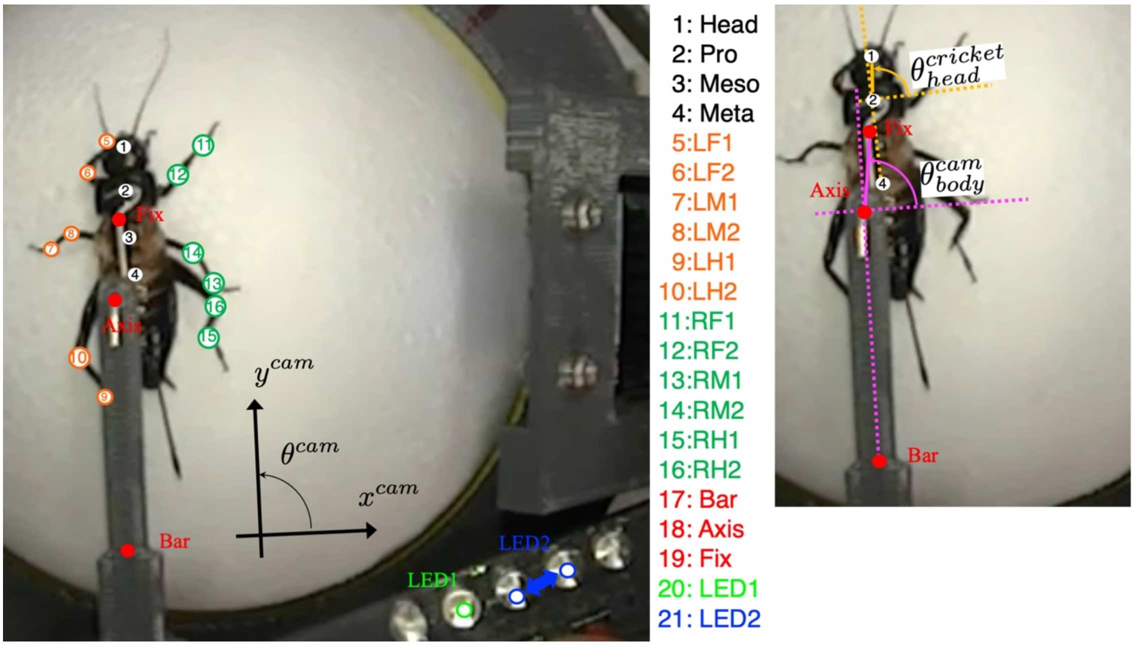

Fig. S2: Marker positions for pose estimation during walking by using DeepLabCut (DLC) [39,40]. The marker numbers are shown in the middle. By using the vertical positions of leg joints on each leg, we calculated power and recovery stroke (stance and swing phase) on leg movements, results in the quantification of gait diagrams as shown in Fig. 1. As shown in the right-hand area, the head angle in the cricket frame was calculated by using the cricket body positions (Head:1, Pro:2, and Meta:4) and the body angle in the camera frame was calculated by using the frame positions (Fix:19, Axis:18, and Bar:17). LED2 position was used for the data synchronization with the optical flow sensors.

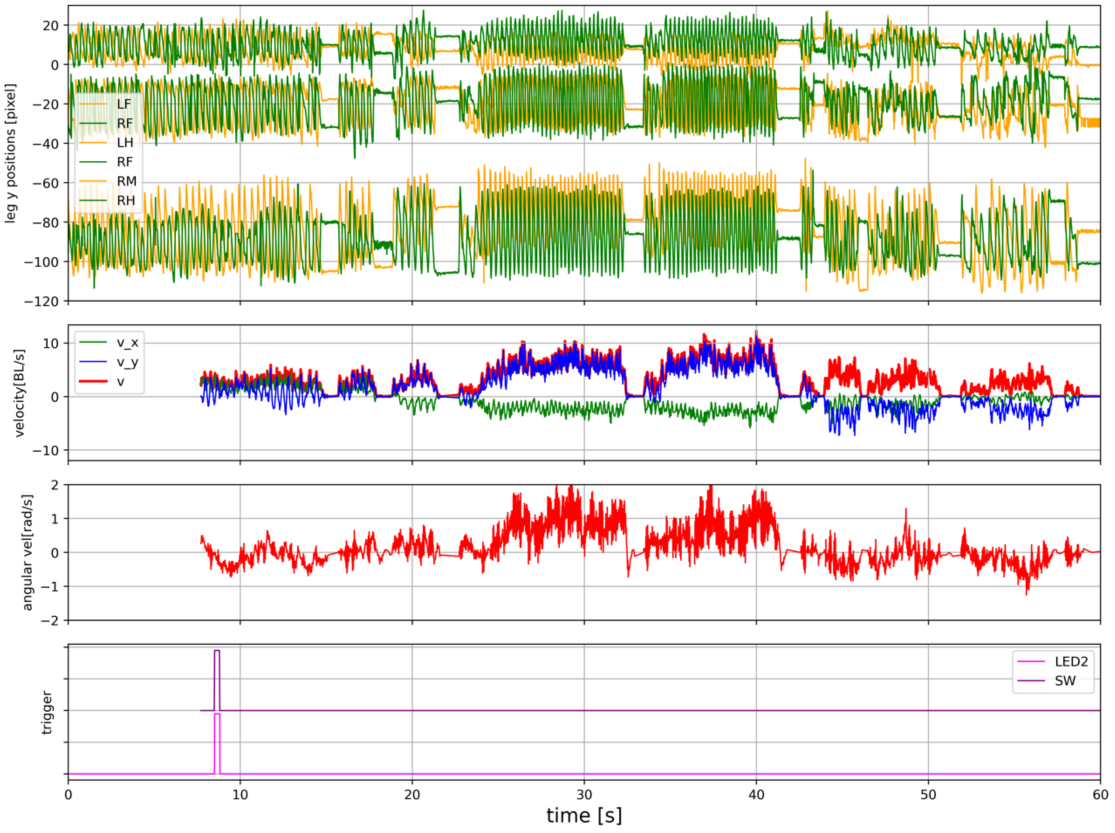

Fig. S3: Synchronisation of estimated marker positions by DLC and walking velocities calculated the optical flow sensors. The bottom panel shows the trigger signals: Magenta color signal shows the estimated position of LED2 (Fig.S2); and purple color signal shows the recorded tactile switch signal for LED2 in the Raspberry Pi. The top panel show the DLC estimated leg y position date for each leg (Orange lines show the left legs and green lines show the right legs, higher position shows the cranial side (head) of the body). The second (green: x velocity, blue: y velocity, and red: velocity on the walking direction) and third panels (angular velocity ω_{yaw} of body rotation on the treadmill) from the top show the velocity data obtained by the optical flow sensors. By synchronizing the triggers, we could analyse the leg position data and walking velocity data simultaneously.

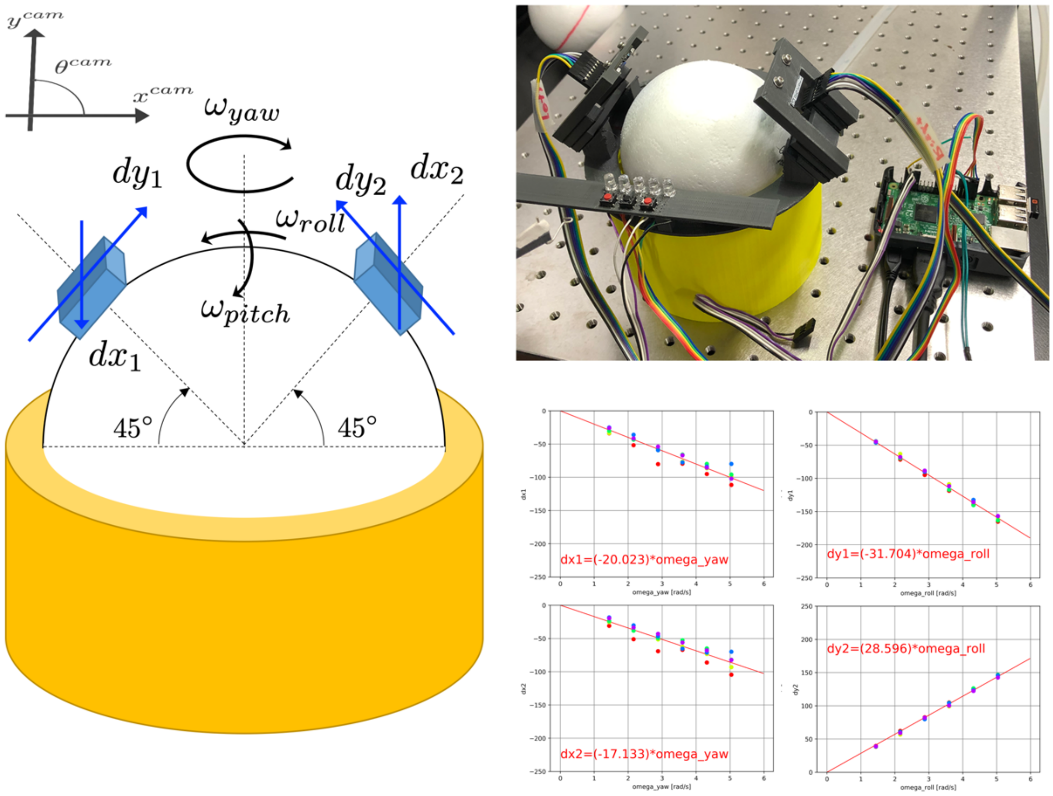

Fig. S4: Measurement system of walking velocity and angular velocity of body rotation by using 2 optical flow sensors [31,38]. Left figure shows relation between the camera and sphere treadmill coordinates on the measurement system, where the sensors located just above the sphere. Right lower figure shows the calibrated relationship between angular velocity of the spherical rotation and detected sensor value. The horizontal axis indicates the angular velocity of spherical rotation ω_{yaw}, ω_{roll} and the vertical axis indicate the corresponding sensor value (we measured sensor values on 5 times with the same conditions on 6 angular velocities). In this calibration, we used a precise controlled electrical motor (DYNAMIXEL XH430-W210-R, ROBOTIS, INC.) to get the σ parameters in Eqs. (2)-(4). The motor can be controlled by using Raspberry Pi with the continuous and constant rotation command. Then, by attaching the same sphere to the motor and rotate it on the treadmill base under the change of rotational speed, we measured the sensor value under the constant speed spherical rotation in the yaw and roll direction.

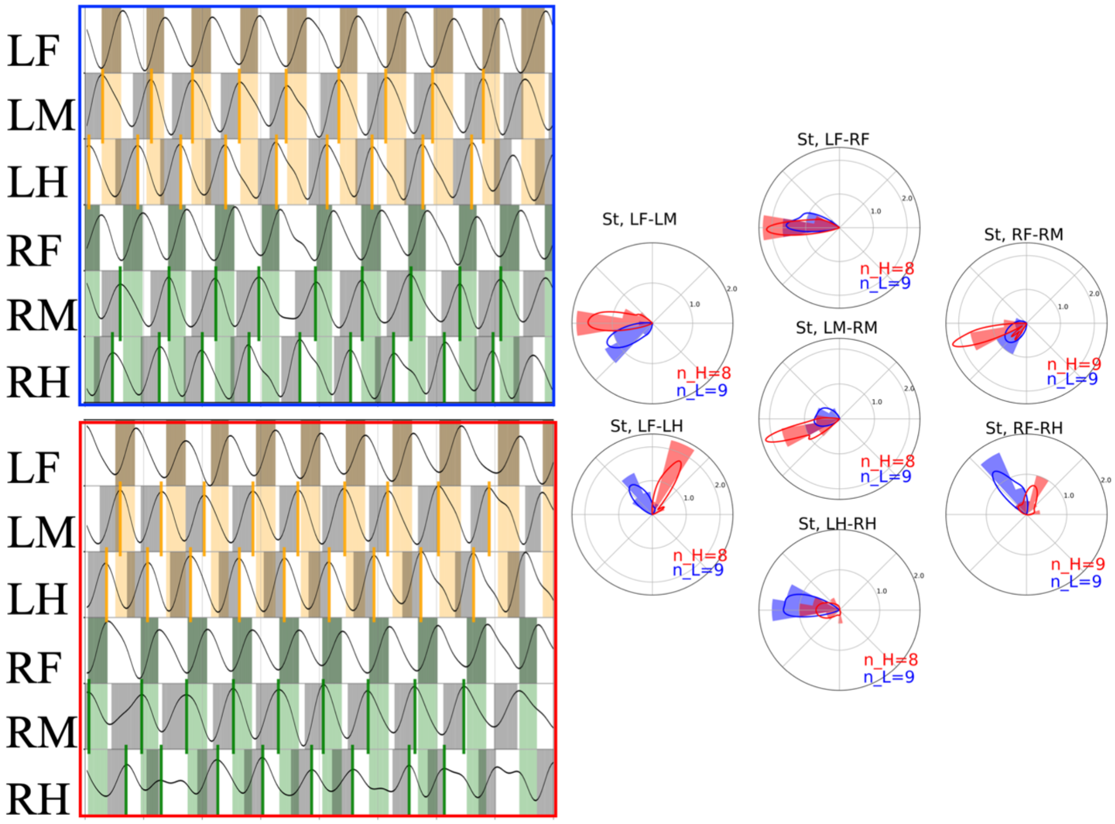

Fig. S5A: Gait pattern of the cricket (all data in 2 s intervals, blue and red colours represent gait data of low and high walking velocities, respectively). A Intact, blue = 2.85 BL(Body Length)/s, red = 6.22 BL/s; The left panels show gait diagrams in walk, representative of each condition. The black lines show normalized leg joint trajectory of each leg estimated by DLC. The grey coloured region indicates swing phase (recovery stroke) periods, in which a leg moves forward. The orange and green coloured region indicate the swing phase of LF and RF legs. Bold lines (LM and LH: orange, RM and RH: green) on the gait diagrams show the timing of foot contact on legs for visibility. The right panels show phase difference between the foot contact timings of legs using circular histogram and kernel density estimation (KDE) plots (left top: LF-LM, left bottom: LF-LH, centre top: LF-RF, centre middle: LM-RM, centre bottom: LH-RH, right top: RF-RM, right bottom: RF-RH).

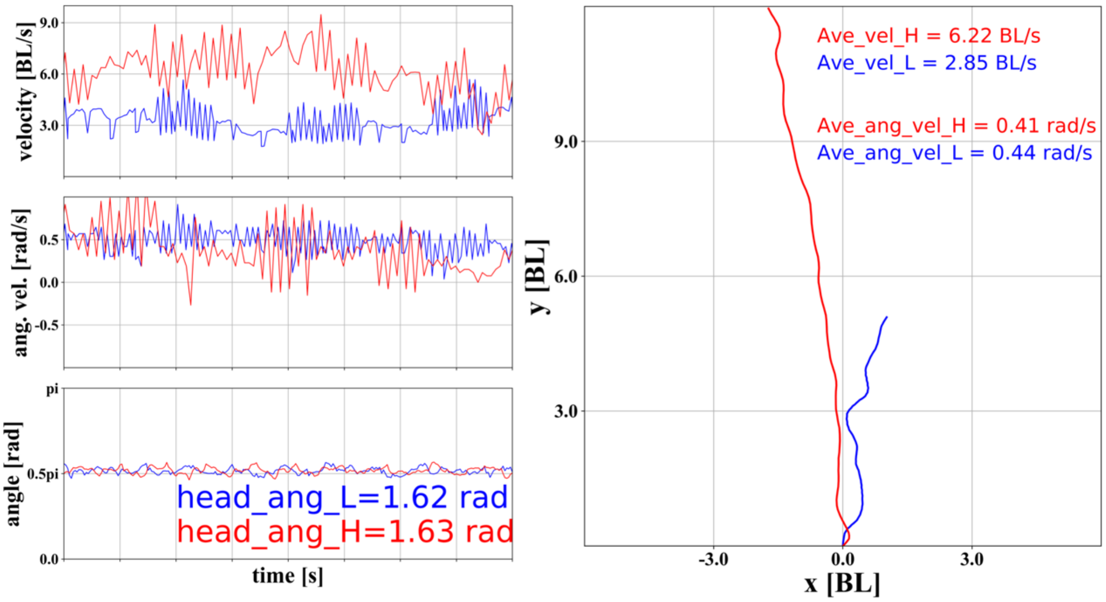

Fig. S5B: Gait pattern of the cricket (all data in 2 s intervals, blue and red colours represent gait data of low and high walking velocities, respectively). A. Intact, blue = 2.85 BL(Body Length)/s, red = 6.22 BL/s; The left panels from the left show measured walking velocities [BL/s] in the walking direction (top), angular velocities [rad/s] of body rotation in the yaw axis (middle), and head angles [rad] (bottom) during walking. The right panels show walking trajectories in the x-y plane [BL], which are integrated over the 2 s from the origin (0, 0) by using velocity, angular velocity, and body angle data for each condition.

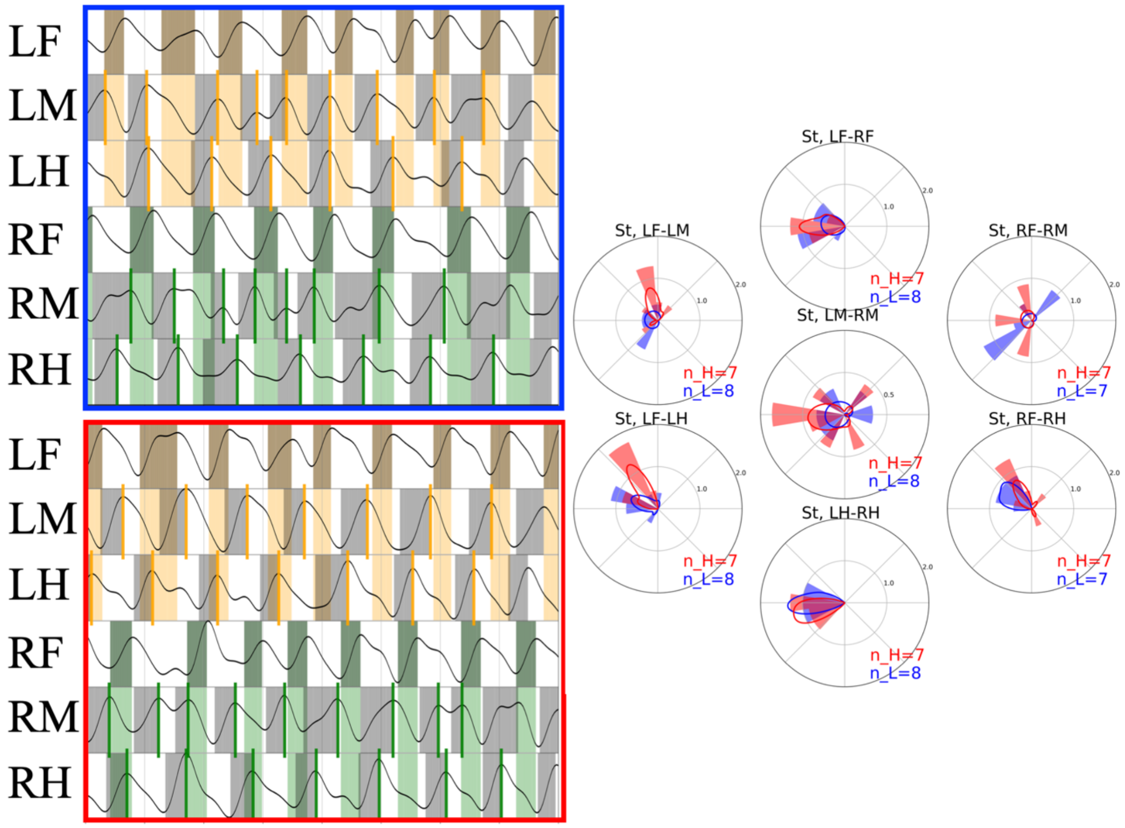

Fig. S6A: Gait pattern of the cricket (all data in 2 s intervals, blue and red colours represent gait data of low and high walking velocities, respectively). B. Both middle legs amputated at the femur-tibial (FTi) joints, blue = 2.98 BL/s, red = 5.28 BL/s; The left panels show gait diagrams in walk, representative of each condition. The black lines show normalized leg joint trajectory of each leg estimated by DLC. The grey coloured region indicates swing phase (recovery stroke) periods, in which a leg moves forward. The orange and green coloured region indicate the swing phase of LF and RF legs. Bold lines (LM and LH: orange, RM and RH: green) on the gait diagrams show the timing of foot contact on legs for visibility. The right panels show phase difference between the foot contact timings of legs using circular histogram and kernel density estimation (KDE) plots (left top: LF-LM, left bottom: LF-LH, centre top: LF-RF, centre middle: LM-RM, centre bottom: LH-RH, right top: RF-RM, right bottom: RF-RH).

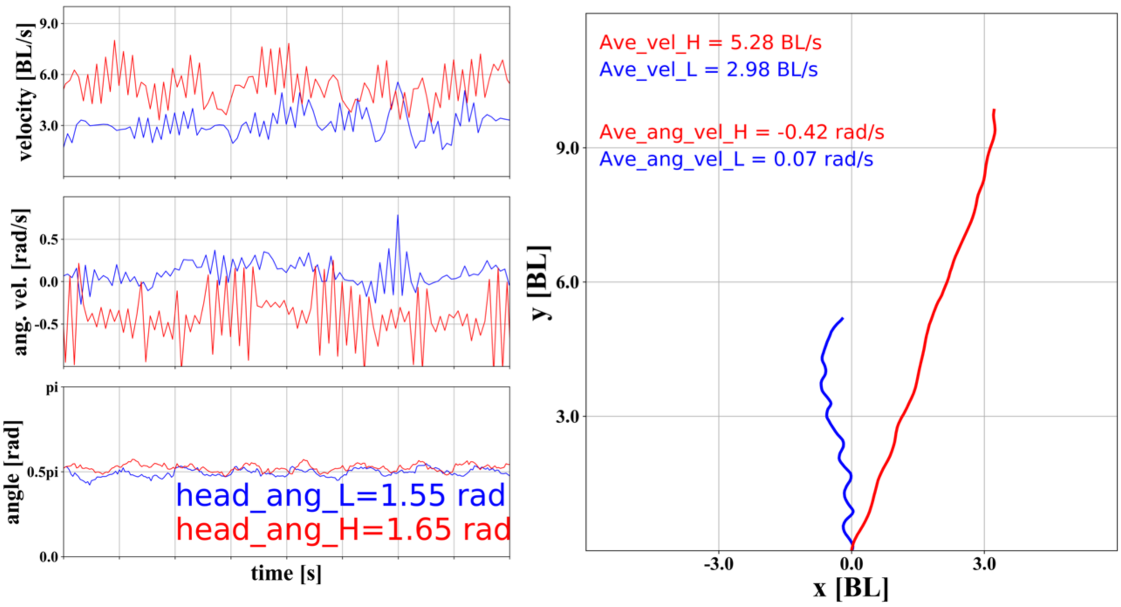

Fig. S6B: Gait pattern of the cricket (all data in 2 s intervals, blue and red colours represent gait data of low and high walking velocities, respectively). B. Both middle legs amputated at the femur-tibial (FTi) joints, blue = 2.98 BL/s, red = 5.28 BL/s; The left panels from the left show measured walking velocities [BL/s] in the walking direction (top), angular velocities [rad/s] of body rotation in the yaw axis (middle), and head angles [rad] (bottom) during walking. The right panels show walking trajectories in the x-y plane [BL], which are integrated over the 2 s from the origin (0, 0) by using velocity, angular velocity, and body angle data for each condition.

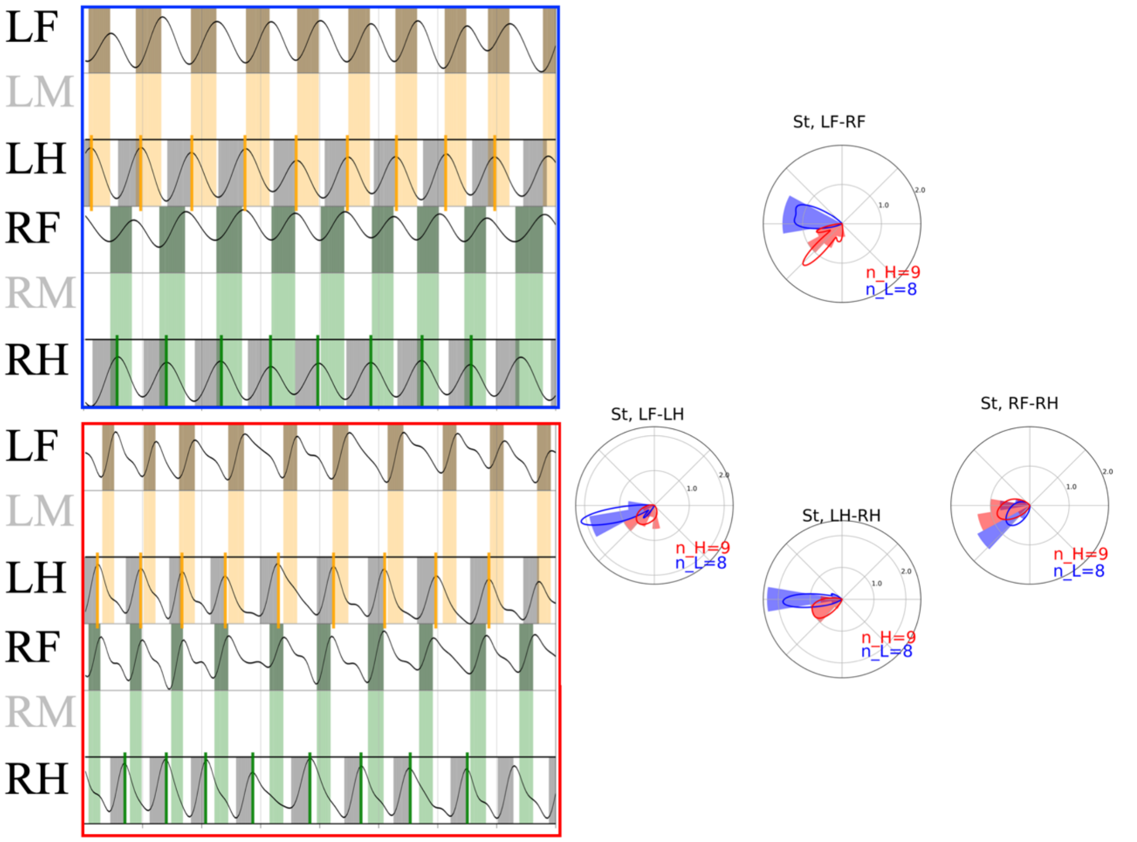

Fig. S7A: Gait pattern of the cricket (all data in 2 s intervals, blue and red colours represent gait data of low and high walking velocities, respectively). C. Both middle legs amputated at the coxatrochanteral joint (CTr) joints, blue = 2.38 BL/s, red = 4.75 BL/s.The far left panels show gait diagrams in walk, representative of each condition. The black lines show normalized leg joint trajectory of each leg estimated by DLC. The grey coloured region indicates swing phase (recovery stroke) periods, in which a leg moves forward. The orange and green coloured region indicate the swing phase of LF and RF legs. Bold lines (LM and LH: orange, RM and RH: green) on the gait diagrams show the timing of foot contact on legs for visibility. The right panels show phase difference between the foot contact timings of legs using circular histogram and kernel density estimation (KDE) plots (left top: LF-LM, left bottom: LF-LH, centre top: LF-RF, centre middle: LM-RM, centre bottom: LH-RH, right top: RF-RM, right bottom: RF-RH).

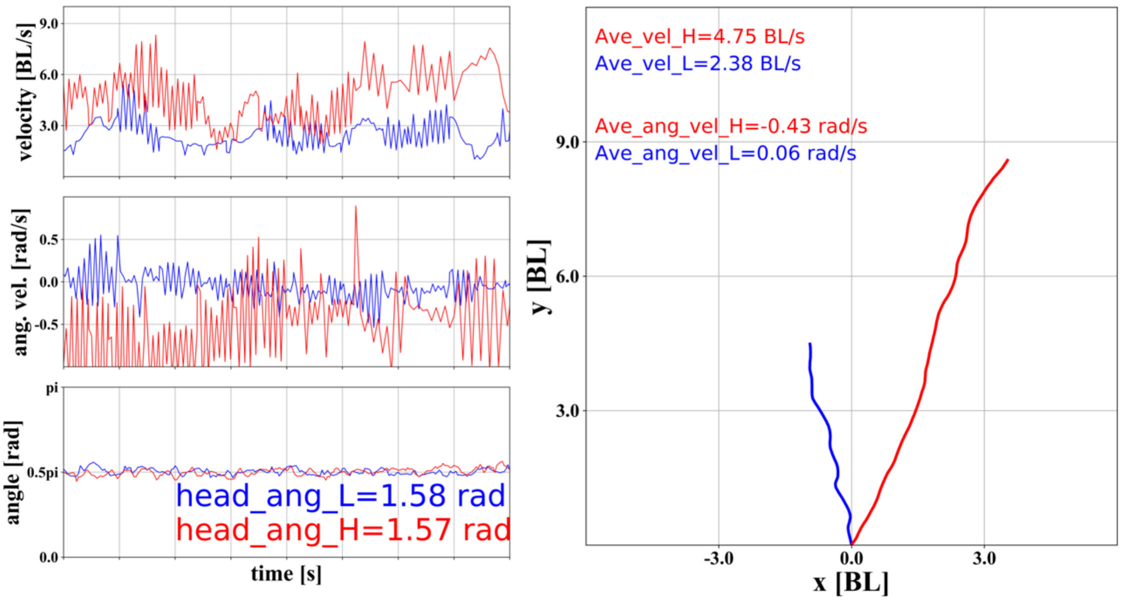

Fig. S7B: Gait pattern of the cricket (all data in 2 s intervals, blue and red colours represent gait data of low and high walking velocities, respectively). C. Both middle legs amputated at the coxatrochanteral joint (CTr) joints, blue = 2.38 BL/s, red = 4.75 BL/s. The left panels show measured walking velocities [BL/s] in the walking direction (top), angular velocities [rad/s] of body rotation in the yaw axis (middle), and head angles [rad] (bottom) during walking. The right panels show walking trajectories in the x-y plane [BL], which are integrated over the 2 s from the origin (0, 0) by using velocity, angular velocity, and body angle data for each condition.

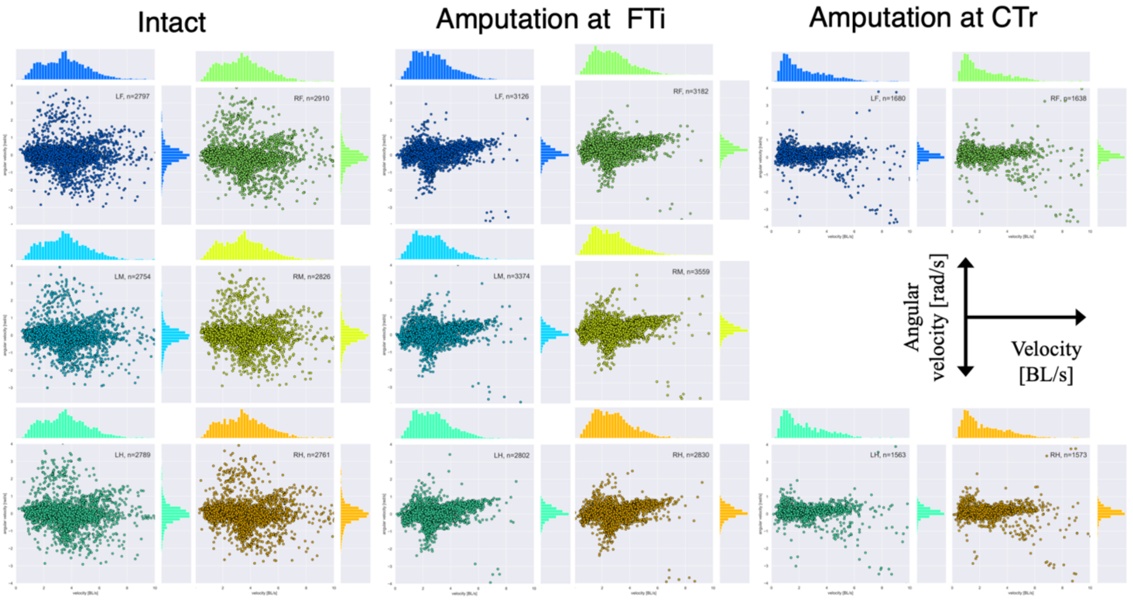

Fig. S8A: Quantification of obtained data: walking velocity [BL/s] and angular velocity of body rotation [rad/s] on each period for all data. Left: Intact walking, Center: Both middle legs are amputated at the FTi joints, Right: Both middle legs are amputated at the CTr joints. Each panel shows the data on the corresponding leg (upper left: LF, center left: LM, lower left: LH, upper right: RF, center right: RM, lower right: RH).

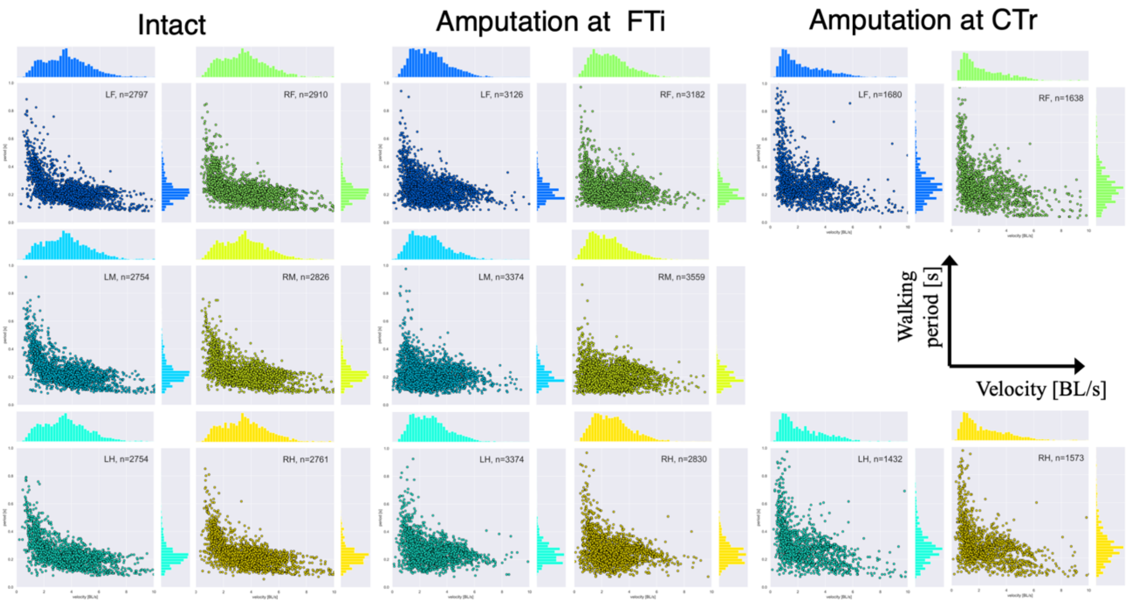

Fig. S8B: Quantification of obtained data: walking velocity versus walking period for each period. Left: Intact walking, Center: Both middle legs are amputated at the FTi joints, Right: Both

middle legs are amputated at the CTr joints. Each panel shows the data on the corresponding leg (upper left: LF, center left: LM, lower left: LH, upper right: RF, center right: RM, lower right: RH). The data confirmed the validity of measurements and analyses of our setups due to the general trends[13,15,42] on the insect locomotion: walking period decreases with the increase of locomotion speed.

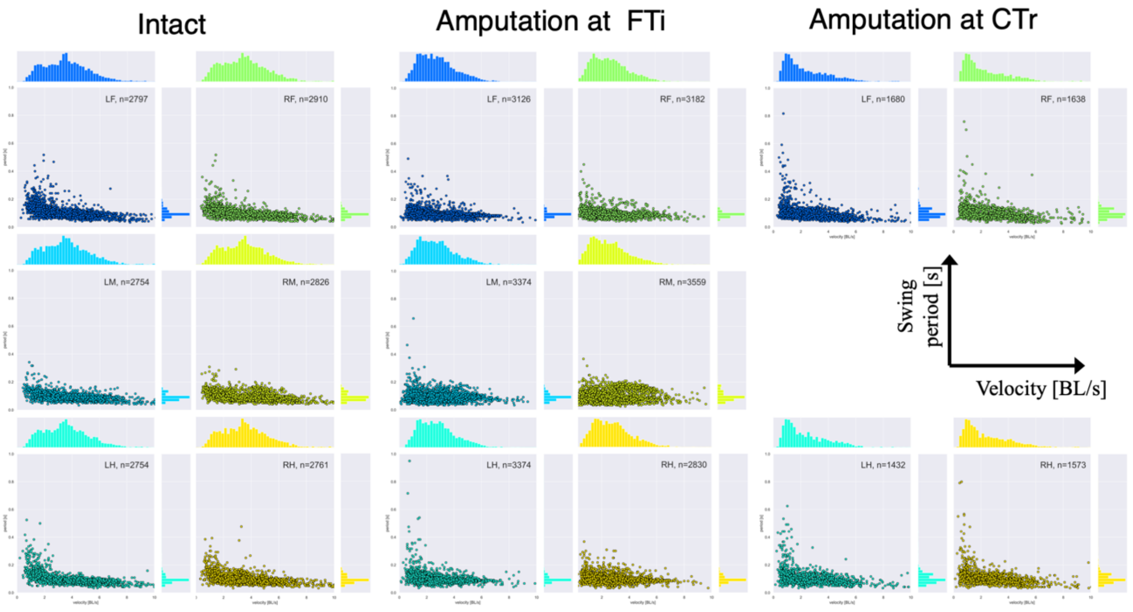

Fig. S8C: Quantification of obtained data: walking velocity versus swing phase (recovery stroke) period for each period. Left: Intact walking, Center: Both middle legs are amputated at the FTi joints, Right: Both middle legs are amputated at the CTr joints. Each panel shows the data on the corresponding leg (upper left: LF, center left: LM, lower left: LH, upper right: RF, center right: RM, lower right: RH). The data confirmed the validity of measurements and analyses of our setups due to the general trends[13,15,42] on the insect locomotion: walking period decreases with the increase of locomotion speed, whereas swing phase period (recovery stroke) remains almost same period region even if the speed increase.

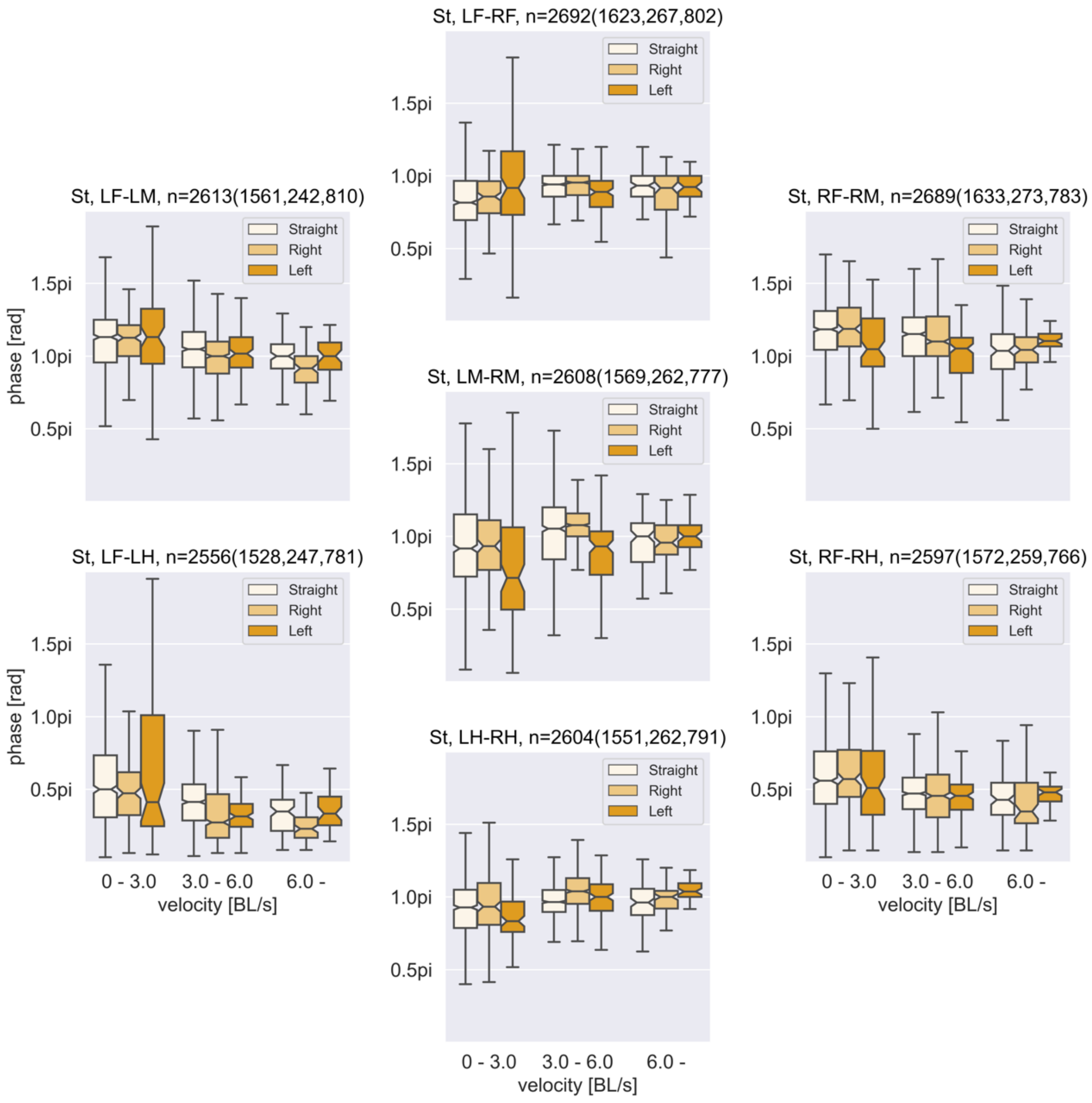

Fig. S9: Effect of body rotation on leg coordination. Each panel shows phase differences between foot contact timings (from swing to stance phases) of legs (left top: LF-LM, left bottom: LF-LH, centre top: LF-RF, centre middle: LM-RM, centre bottom: LH-RH, right top: RF-RM, right bottom: RF-RH). *n* indicates the analysed periods for each panel (n=All (Straight, Right, Left)). The colour difference in each pane represents walking direction: Straight = -0.5 to 0.5 rad/s, Left > 0.5 rad/s, Right < -0.5 rad/s. Obviously, comparing with Fig. 2 A, the difference among Straight, Right, and Left condition become smaller.

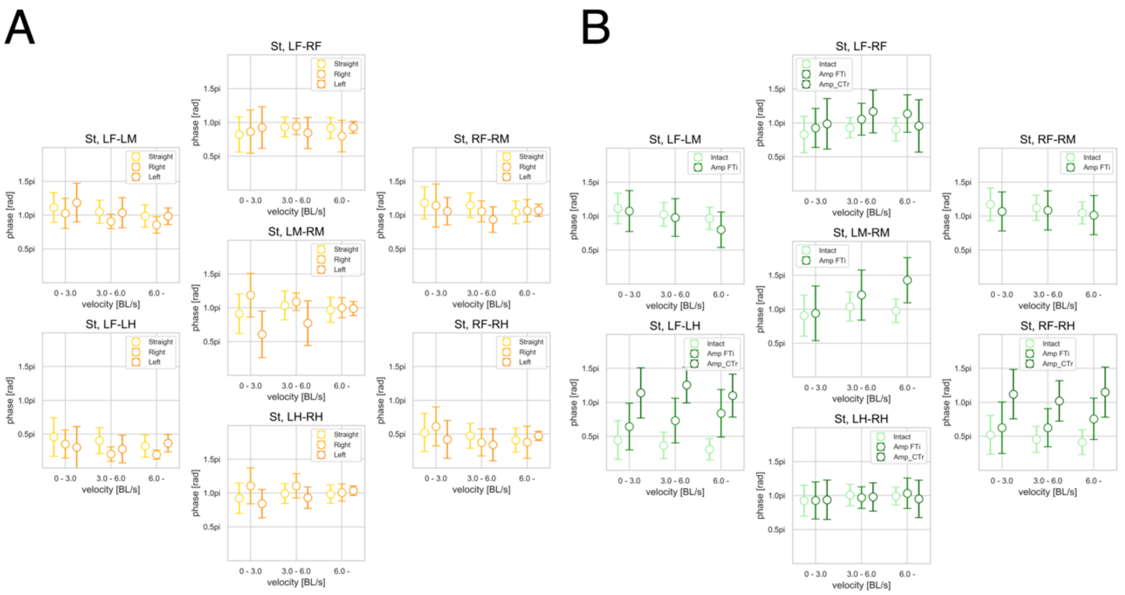

Fig. S10: Circular statistics (average and standard deviation of phase data) [53] on Fig 2. The same trends confirmed the validity of the statistical data in this study.

Table S1: Body length of crickets we used on the experiment of setup 1.

Cricket No.06, male, BL = 23.80 mm.
Cricket No.07, female, BL = 24.39 mm.
Cricket No.08, female, BL = 23.56 mm.
Cricket No.09, male, BL = 21.88 mm.
Cricket No.11, female, BL = 22.51 mm.
Cricket No.12, male, BL = 21.44 mm.

Reference

S1. Furukawa, N, Tomioka, K., & Yamaguchi, T. Functional anatomy of the musculature and innervation of the neck and thorax in the cricket, *Gryllus bimaculatus* (in Japanese), *Zool. Mag.* **92**, 371-385 (1983).
